# Improved Antibacterial Properties of Fermented and Enzymatically Hydrolyzed Bee Pollen and Its Combined Effect with Antibiotics

**DOI:** 10.3390/ph18010015

**Published:** 2024-12-26

**Authors:** Vaida Damulienė, Vilma Kaškonienė, Paulius Kaškonas, Rūta Mickienė, Audrius Maruška

**Affiliations:** 1 Instrumental Analysis Open Access Centre, Vytautas Magnus University, LT-44404 Kaunas, Lithuania; vaida.damuliene@vdu.lt (V.D.); ruta.mickiene@vdu.lt (R.M.); audrius.maruska@vdu.lt (A.M.); 2 Institute of Metrology, Kaunas University of Technology, LT-51368 Kaunas, Lithuania; paulius.kaskonas@ktu.lt

**Keywords:** European bee pollen, antimicrobial activity, solid-state fermentation, *Viscozyme^®^ L*, *Clara-diastase*, cellulase

## Abstract

**Background/Objectives:** A variety of phytochemicals from different plants are collected by bees into bee pollen granules. This research focused on evaluating the effects of lactic acid fermentation and enzymatic hydrolysis on the antibacterial activity of bee pollen and its interaction with antibiotics. There is limited knowledge regarding the interactions between treated bee pollen extracts and antibiotics, and this study contributes to the field by providing new insights into the antibacterial activity of pollen subjected to eight distinct treatment methods. **Methods**: Bee pollen’s bacterial fermentation using a *Lacticaseibacillus rhamnosus* culture and spontaneous fermentation were performed. Bee pollen hydrolysis was performed using commercial enzymes, including enzyme mixtures as well as pure enzymes. The agar well diffusion assay was employed to assess the antibacterial activity against *Staphylococcus aureus*, *Listeria monocytogenes*, and *Salmonella enterica* serovars Enteritidis and Typhimurium, as well as their interaction with antibiotics (ceftazidime, ciprofloxacin, oxytetracycline dihydrate, and erythromycin). **Results**: This study showed an enhancement in bee pollen’s antibacterial activity after both fermentation and enzymatic hydrolysis. The increase varied with the pollen’s origin, treatment type, and culture used for antimicrobial tests. More than 77% of bee pollen extracts demonstrated a synergistic effect with antibiotics across all tested bacterial strains, while antagonistic interactions were comparatively rare. **Conclusions**: The applied treatment methods can improve the antibacterial properties of bee pollen. Bee pollen extracts, in combination with antibiotics, can enhance their effectiveness. These findings provide new insights into the potential use of bee pollen in combating bacterial infections.

## 1. Introduction

Since ancient times, many diseases have been treated using medicines made from natural products. Over the years, many of these have been replaced by synthetic chemicals; nevertheless, interest in natural substances is growing again [1]. Various pharmaceutical companies are conducting increasing research to discover new sources of biologically active compounds and evaluate their properties. Detailed studies have determined that the resistance of pathogenic microorganisms to antimicrobial agents is rapidly increasing, prompting the search for new therapeutic compounds that not only have healing properties but also fewer harmful side effects [2].

A significant portion of published studies relate to evaluating the positive biological properties or chemical components of various plants or their parts. Plants are among the best sources of natural products, especially various bioactive compounds [3,4]. These extracts can be used as antimicrobial agents to treat diseases caused by various pathogenic organisms. Furthermore, most of them exhibit active pharmacological effects, such as antibacterial, antifungal, antiparasitic, or anticancer properties [5,6,7,8,9].

The diverse chemical composition of plants and their parts, especially biologically active substances extracted from essential oils, increasingly encourages the investigation of the synergistic effects of plant extracts with antibiotics, which can help reduce the quantities of pharmaceutical drugs used [10,11]. Based on the available data in the literature, it has been reported that only a specific subset of plant extracts enhances the effectiveness of antibiotics [12]. Furthermore, the combination of synthetically derived and natural compounds not only leads to fewer side effects on living organisms but also contributes to reducing bacterial resistance.

It is not surprising that bee-collected pollen also exhibits various biological activities, as it is part of the plant that worker bees collect and mix with nectar and salivary secretions [13]. Bee pollen in the recent decade was re-discovered, and many studies revealing their impact on human health as food or a natural source remedy indicate its potential in promoting health, preventing disease, and improving quality of life [14,15,16]. Bee pollen is one of the most easily accessible natural products receiving considerable attention in the field of pharmaceutical applications. Its diverse chemical composition contributes to its potent antibacterial, antifungal, anti-inflammatory, and anticancer properties [15,16]. Studies have identified rutin, vanillic acid, gallic acid, quercetin, p-coumaric acid, hesperidin, kaempferol, apigenin, luteolin, and isorhamnetin as the main compounds responsible for these properties. More detailed analysis of these compounds revealed their effectiveness against specific pathogenic organisms, such as *S. aureus*, *E. coli*, *Streptococcus viridans*, and *Pseudomonas aeruginosa* [17,18]. Moreover, the antimicrobial activity of bee pollen is exceptionally influenced by highly active lactic acid bacteria. The uniqueness of these bacteria lies in the production of specific substances called bacteriocins, which halt the growth and development of pathogenic bacteria by disrupting their membranes [19]. The activity of bacteriocins produced by lactic acid bacteria against *Listeria monocytogenes*, *Listeria innocua*, *S. aureus*, *E. coli*, *Micrococcus luteus*, *P. aeruginosa*, *B. subtilis*, and *Salmonella* serovars Enteritidis and Typhimurium has been proven [20,21].

Bee pollen is one of the most popular natural products used commercially. However, its outer wall, composed of sporopollenin, is exceptionally resilient, elastic, chemically resistant, and provides protection from external damage. Consequently, bee pollen has limited bioavailability due to difficulties in digestion by humans [22,23,24]. Therefore, it is crucial to discover straightforward ways to enhance its bioavailability [23,25]. Mechanical treatment involving shear forces that generate heat [26], physical treatment using supercritical fluids [27], lactic acid fermentation [28,29,30], or enzymatic hydrolysis [31,32] have been studied for this purpose.

The available literature on the impact of bee pollen on antibiotic efficacy is scarce. Honey and propolis are the most frequently tested bee products in combination with antibiotics [33,34]. There are only a few studies investigating the use of bee pollen as an enhancer of drug effects. For example, research by Hanafy et al. [35] suggests that bee pollen can synergize with chemotherapy drugs, specifically Avastin^®^ (bevacizumab), enhancing their anti-cancer efficacy. Extracts of bee pollen phenolic compounds and nanoparticles have shown enhanced anti-proliferative activity against cancer cell lines, such as MCF-7 breast cancer cells and A549 lung cancer cells, when combined with these drugs. This synergy likely arises from bee pollen’s high flavonoid and polyphenol content, which contributes to cancer cell apoptosis and the inhibition of cancer-promoting signaling pathways. Araque and Vit [36] highlighted the antimicrobial potential of bee pollen and its ability to synergize with conventional treatments after analyzing *Tetragonisca angustula* (stingless bee) pot-pollen in combination with amikacin and meropenem against extensively drug-resistant bacteria. Additionally, the study by Ilie et al. [37] demonstrated a synergistic inhibitory effect when bee pollen extracts were combined with soluble compounds from two lactic acid bacterial strains, *L. rhamnosus* MF9 and *E. faecalis* 2M17, against two clinical pathogenic bacteria.

These studies reveal the potential of bee pollen extracts and suggest that interactions between bee pollen and antibiotics or other drugs can vary, depending on the specific application, the organism involved, the botanical origin of the bee pollen, or even the treatment method used, as demonstrated in the present study.

While bee pollen is valued for its antibacterial properties and high nutritional content, it is important to acknowledge that bees may also bring hazardous materials into the hive from the fields. These materials include pesticides, pyrrolizidine alkaloids, heavy metals, and even mycotoxins. Currently, European Commission regulations on bee pollen safety are less stringent than those for honey, likely due to its lower consumption levels. However, scientific studies confirm that the presence of these contaminants in bee pollen is a significant concern [38,39]. Existing regulations, such as EU 2023/915 [40] and EC 366/2005 [41], primarily focus on honey. For bee pollen, safety regulations are limited to specific pesticides and pyrrolizidine alkaloids.

There is optimism that in the future, potential risks associated with pesticides, pyrrolizidine alkaloids, heavy metals, and mycotoxins in bee pollen will be more comprehensively addressed and corresponding regulations will be improved. Therefore, when considering the practical application of bee pollen in health, pharmacy, or the food industry, it is essential to first ensure that the bee pollen used is free from contaminants. The aim of this research was to evaluate the effects of solid-state lactic acid fermentation and enzymatic hydrolysis on the antibacterial activity of bee pollen and its interaction with antibiotics.

## 2. Results and Discussion

**Figure 1:**
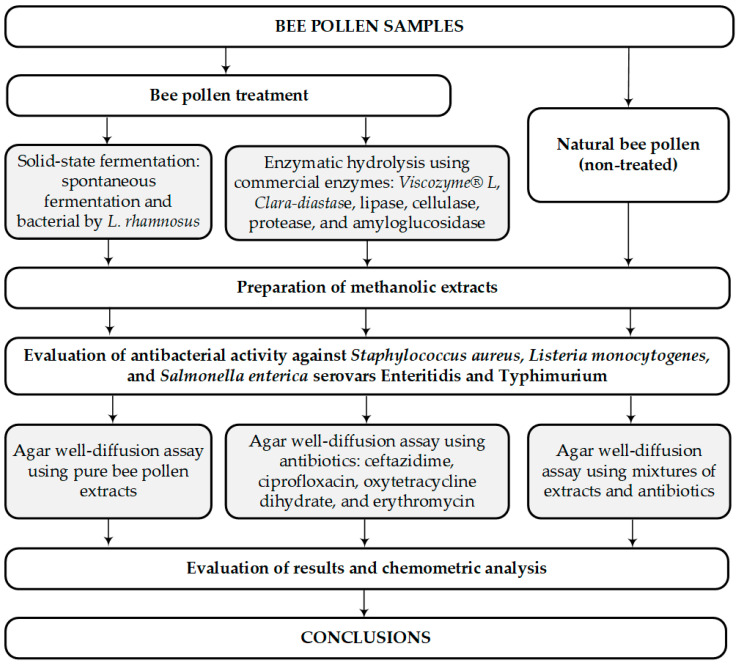
Design of experimental research.

Nine samples of bee pollen from different regions of Europe were subjected to spontaneous fermentation, bacterial fermentation using *L. rhamnosus* culture, and enzymatic hydrolysis with *Clara-diastase*, *Viscozyme^®^ L*, lipase, protease, amyloglucosidase, and cellulase. The enzymes were selected based on their ability to degrade certain structural components of the pollen wall present in the exine, intine, or both [42,43]. The impact of fermentation on antibacterial activity against *Staphylococcus aureus*, *Listeria monocytogenes*, and *Salmonella enterica* serovars Enteritidis and Typhimurium was evaluated. Additionally, in vitro interactions of natural, fermented, and enzymatically hydrolyzed bee pollen in combination with antibacterial agents were assessed against each bacterial strain analyzed. Antibiotics from different classes were selected for the study based on their chemical structure: cephalosporins (ceftazidime pentahydrate), fluoroquinolones (ciprofloxacin), tetracyclines (oxytetracycline), and macrolides (erythromycin). The experimental design is presented in Figure 1.

First of all, to determine the impact of methanol on antibacterial activity, the antibacterial activity of 80% methanol alone was independently evaluated as part of this study. The results indicated that the effect of methanol was negligible, with the clear zone radius around the wells not exceeding 1 mm (the highest average clear zone was 0.75 mm). Additionally, the extracts were diluted with water at a 1:1 ratio or mixed with an aqueous antibiotic solution before analysis, effectively reducing the concentration of methanol by half. Therefore, the impact of methanol on the observed antibacterial activity was considered minimal and unlikely to interfere with the findings. This conclusion aligns with the literature data showing that methanol has weak bactericidal properties compared to other alcohols [44]. Methanol is rarely used as a disinfectant in healthcare settings due to its limited effectiveness against microorganisms [45].

### 2.1. Antimicrobial Activity of Treated Bee Pollen

The results of the antimicrobial activity of differently treated and untreated bee pollen extracts, as well as their combinations with antibiotics, are presented in Table 1 and expressed as the average diameter of the inhibition zone in millimeters.

A positive effect of both solid-state fermentation and enzymatic hydrolysis on bee pollen antibacterial activity was observed. Antibacterial activity after spontaneous fermentation significantly (*p* ≤ 0.05) increased by 1.04–2.18 times against *S. aureus*, 1.09–1.38 times against *L. monocytogenes*, 1.18–2.22 times against *Salmonella* Enteritidis, and 1.07–2.92 times against *Salmonella* Typhimurium. Antibacterial activity after bacterial fermentation significantly (*p* ≤ 0.05) increased by 1.18–2.31 times against *S. aureus*, 1.21–1.61 times against *L. monocytogenes*, 1.26–2.34 times against *Salmonella* Enteritidis, and 1.21–4.08 times against *Salmonella* Typhimurium. Higher antibacterial activity compared to natural or spontaneously fermented bee pollen was observed after bacterial fermentation with *L. rhamnosus*. These results may be influenced by the superior breakdown of the pollen cell wall by *L. rhamnosus* or its capability to release bacteriocins, as suggested by some studies [46].

Moreover, a significant intensification of antibacterial activity was observed after enzymatic hydrolysis of bee pollen against cultures of pathogenic bacteria. Antibacterial activity showed statistically significant (*p* ≤ 0.05) differences in all enzymatically hydrolyzed bee pollen samples collected from different regions of Europe. Overall, the antibacterial efficacy following enzymatic hydrolysis showed a significant (*p* ≤ 0.05) increase in activity by 1.14–6.42 times against *S. aureus*, 1.05–3.31 times against *L. monocytogenes*, 1.04–6.06 times against *Salmonella* Enteritidis, and 1.04–7.56 times against *Salmonella* Typhimurium. The most significant increases in antibacterial activity were observed after enzymatic hydrolysis with *Clara-diastase* (1.09 to 5.98 times) and *Viscozyme^®^ L* (1.21 to 6.50 times), while the smallest increases were determined with amyloglucosidase (1.04 to 3.51 times). The substantial increases in antibacterial activity following enzymatic hydrolysis reveal that it would be advisable to treat bee pollen enzymatically with *Clara-diastase* and *Viscozyme^®^ L* before use in any applications where antibacterial properties are desired, such as in food preservation, natural health supplements, cosmetic formulations, or antimicrobial coatings for packaging materials.

By comparison, the antimicrobial activity of natural (spontaneous) fermentation and enzymatic hydrolysis can be observed to depend on the type of bee pollen, the enzyme used, and the bacterial strain tested. However, in most cases, enzymatic hydrolysis resulted in better antimicrobial activity compared to spontaneous fermentation. For instance, bee pollen extracts hydrolyzed with cellulase showed 1.2 to 4.3 times stronger inhibition, which was statistically significant (*p* ≤ 0.05), compared to those subjected to spontaneous fermentation. Similarly, extracts treated with enzyme mixtures such as *Viscosyme*^®^ *L* and *Clara-diastase* exhibited higher antimicrobial activity (1.1–3.7 times), with a few exceptions where the difference was not statistically significant (*p* ≤ 0.05). In contrast, for *Salmonella* Enteritidis inhibition, extracts from spontaneous fermentation demonstrated 1.2–1.9 times higher inhibition, which was statistically significant (*p* ≤ 0.05), compared to pollen hydrolyzed with lipase or amyloglucosidase in 12 out of 18 cases; in the remaining cases, the results were not statistically different (*p* ≤ 0.05). In summary, while the antimicrobial activity of bee pollen increased following both spontaneous fermentation and enzymatic hydrolysis, the highest activity was generally observed with enzymatic hydrolysis, particularly when cellulase was used. However, it is important to note that the differences can vary depending on the specific conditions.

Variations in the obtained results suggest that geographical origins, likely closely linked to the botanical origin of bee pollen, as well as climatic conditions, play an extremely important role in the antibacterial activity of natural or treated pollen. The palynological analysis was not performed in this study; however, a visual inspection of the bee pollen indicates that the samples have a diverse range of colors, which suggests the presence of pollen from different botanical origins (Figure S1). The most significant differences in antibacterial activity against both *Salmonella* Enteritidis and *L. monocytogenes* were observed in pollen from Lithuania, Sweden, and Slovakia when comparing the results obtained after bacterial fermentation and spontaneous fermentation. The representation of antibacterial activity depending on bee pollen’s geographical origin has been added to the supplementary file (Table S1). In contrast, the differences in antibacterial activity between different fermentation types were not substantial in pollen from other countries. However, after spontaneous fermentation, the antibacterial activity against *S. aureus* bacterial cultures in pollen samples from various origins appears to be quite similar, with Lithuanian pollen standing out the most (with activity differences between Lithuanian and other samples reaching 1.5–3.4 times).

The literature highlights that the antibacterial activity of bee pollen has been actively studied in recent years. Kacaniova et al. [47] reported a positive effect of bee pollen from Slovakia on antibacterial activity against *L. monocytogenes*, *P. aeruginosa*, *S. aureus*, *S. enterica*, and *E. coli*. Additionally, studies in various countries have identified antibacterial activity of bee pollen against *S. aureus*, *E. faecalis*, *P. aeruginosa*, *S. enterica*, and *S. pyogenes* [18,37,48,49]. However, the antibacterial activity of fermented bee pollen remains an underexplored area in scientific research by other authors. In our previous study, it was found that the antibacterial activity against *S. aureus* increased by 1.4–2.0 times after spontaneous fermentation of Latvian pollen and by 1.2–2.2 times after fermentation with *L. rhamnosus*. Furthermore, the antibacterial activity of fermented bee pollen was assessed against *M. luteus* and *E. coli* [30].

### 2.2. Antimicrobial Activity of Treated Bee Pollen and Antibiotics Mixtures

This study demonstrated interactions between bee pollen extracts and antibiotics, predominantly synergistic (SY), although some cases showed antagonistic (AN) or additive effects (Table 1). These interactions depended on the treatment method, tested bacteria, antibacterial agent, and the origin of bee pollen, which may be closely correlated with botanical origin and phytochemical composition—both of which were analyzed in this study.

Among the 324 combinations evaluated against *Salmonella* Typhimurium, 261 (80.56%) were synergistic, while 60 (18.52%) showed antagonistic effects. Against *Salmonella* Enteritidis, there were 250 (77.16%) synergistic and 73 (22.53%) antagonistic combinations. For *L. monocytogenes*, 269 (83.02%) combinations were synergistic and 53 (16.36%) were antagonistic. Similarly, against *S. aureus*, 267 (82.41%) combinations were synergistic and 57 (17.59%) were antagonistic. Only six additive interactions across all combinations were detected. It is difficult to determine the best combination of treatment methods and antibiotics. However, it is worth mentioning that the mixtures with bee pollen extracts obtained after spontaneous fermentation, *L. rhamnosus* fermentation, or enzymatic hydrolysis by *Viscozyme^®^ L* showed a synergistic effect, except for the mixtures with erythromycin against *S. aureus* (bee pollen sample was obtained from Malta). Promising results were obtained after blending antibiotics with extracts hydrolyzed by cellulase or *Clara-diastase*, with only 4 antagonistic cases detected out of 171. Plant extracts (and possibly bee pollen as well) can interact with antibiotics through various mechanisms, including changing bacterial cell permeability, blocking bacterial resistance mechanisms like efflux pumps, modifying active sites on bacterial cells, inhibiting enzymes that degrade antibiotics in bacterial cell walls, and preventing bacterial biofilm formation [50]. The antibacterial mechanisms of antibiotics themselves also vary, which may impact their interaction with plant extracts. Antibiotics can inhibit cell wall synthesis, damage the cytoplasmic membrane, hinder nucleic acid and protein synthesis, or target specific enzyme systems [51]. However, further comprehensive studies are necessary to ascertain the functioning of these particular mechanisms. At the moment, the main goal of this study was aimed to evaluate whether the bacterial fermentation or enzymatic hydrolysis has an impact on the antibacterial activity of bee pollen, if there are differences between bee pollen from different geographical origins (geographical region is related to botanical origin), and whether bee pollen may affect the activity of antibiotics.

The strongest effect on *S. aureus* was observed when bee pollen was subjected to enzymatic hydrolysis with *Viscozyme^®^ L* and combined with ceftazidime (15.80 ± 2.45 to 45.90 ± 1.20 mm). The most significant impact on *L. monocytogenes* was noted when bee pollen was enzymatically hydrolyzed with *Clara-diastase* or *Viscozyme^®^ L* and combined with erythromycin (up to 44.85 ± 1.27 and 44.50 ± 1.33 mm, respectively). The highest activity against *Salmonella* Enteritidis was observed when bee pollen underwent bacterial fermentation and was combined with ceftazidime (29.10 ± 0.57 to 45.25 ± 1.05 mm). The greatest effect on *Salmonella* Typhimurium was observed when bee pollen was enzymatically hydrolyzed with *Viscozyme^®^ L* and combined with ceftazidime (9.75 ± 1.16 to 45.40 ± 1.33 mm).

However, antagonistic interactions were detected between antibiotics and bee pollen extracts after enzymatic hydrolysis with lipase, protease, and amyloglucosidase. Antibacterial activity against *S. aureus* decreased by 0.45–0.99 times when bee pollen extract after enzymatic hydrolysis with lipase was combined with antibiotics, against *L. monocytogenes* by 0.46–0.99 times, against *Salmonella* Enteritidis by 0.37–0.98 times, and against *Salmonella* Typhimurium by 0.40–0.99 times. Antibacterial activity against *S. aureus* decreased by 0.46–0.97 times when bee pollen extract after enzymatic hydrolysis with protease was combined with antibiotics, against *L. monocytogenes* by 0.44–0.98 times, against *Salmonella* Enteritidis by 0.22–0.98 times, and against *Salmonella* Typhimurium by 0.38–0.99 times. When bee pollen extracts after hydrolysis with amyloglucosidase were combined with antibiotics, antibacterial activity decreased by 0.31–0.99 times against *S. aureus*, 0.42–0.99 times against *L. monocytogenes*, 0.20–0.99 times against *Salmonella* Enteritidis, and 0.36–0.99 times against *Salmonella* Typhimurium.

According to the literature, the bee pollen wall consists of two layers: the exine (outer) and the intine (inner). The exine is made of polysaccharides and serves as the primary storage for glycoproteins, lipids, and phenols. The intine is composed of cellulose, hemicellulose, and pectin [52]. The structure of the bee pollen wall is more easily broken down by cellulase, *Viscozyme^®^ L* (an enzyme mixture of cellulase, xylanase, arabanase, and glucanase), and *Clara-diastase* (an enzyme mixture of amylase, cellulase, invertase, and peptidase) compared to lipase, protease, or amyloglucosidase due to its main wall components.

From the perspective of bee pollen collection regions, bee pollen from colder climate regions (Lithuania, Sweden, and Poland) demonstrated better antibacterial performance, whereas pollen from southern European countries (Malta, Italy, and Spain) showed lower activity against the tested pathogenic bacteria. The improvement in antibacterial activity after adding antibiotics between samples from Lithuania (highest) and Malta (lowest) reached 75%.

A recent study identified naringenin, quercetin, luteolin, and rutin as the main flavonoids, and ferulic and ellagic acids as the principal phenolic acids, in both natural and fermented bee pollen [28]. Based on the available data in the literature, flavonoids and phenolic acids in bee pollen exhibit antibacterial activity against Gram-negative, Gram-positive bacteria, and even pathogenic fungi [24]. Flavonoids have been shown to potentially enhance the efficacy of antibiotics against pathogenic bacteria by disrupting bacterial cytoplasmic membranes, inhibiting nucleic acid synthesis, or blocking the fatty acid synthesis pathway [5,53]. Meanwhile, ferulic and ellagic acids increase the production of superoxide ions, which disrupt bacterial energy metabolism, alter cell membrane properties, and induce the formation of pores in cell membranes, resulting in leakage of vital intracellular components [54,55].

Some compounds show significant efficacy only when combined with an antibiotic. Identifying the specific compound within an extract responsible for the synergistic interaction is challenging. Additionally, some compounds exhibit synergistic activity through mechanisms beyond their inherent antimicrobial properties due to polyvalent effects [56].

### 2.3. Chemometric Analysis

The acquired measurement data were structured into a data matrix of size of 9 × 4 × 5 × 90, representing the countries of bee pollen collection, the bacteria against which antibacterial activity was assessed, the tested antibiotics, and the treatment ways. Therefore, each tested fermented or enzymatically hydrolyzed bee pollen sample supplemented with antibiotics was characterized by an antibiotic activity profile, composed of 16 variables (4 different antibiotics and 4 different bacteria). The control samples were defined by four variables, elucidating antibacterial activity of the fermented or enzymatically hydrolyzed pollen against tested bacteria without antibiotics.

Prior to statistical analyses, a data standardization procedure was applied to remove bias by subtracting the average value of each variable and scaling the scatter (variance), dividing each variable by its standard deviation. The procedure allowed us to achieve data scattered around a zero value with unified variance.

**Figure 2:**
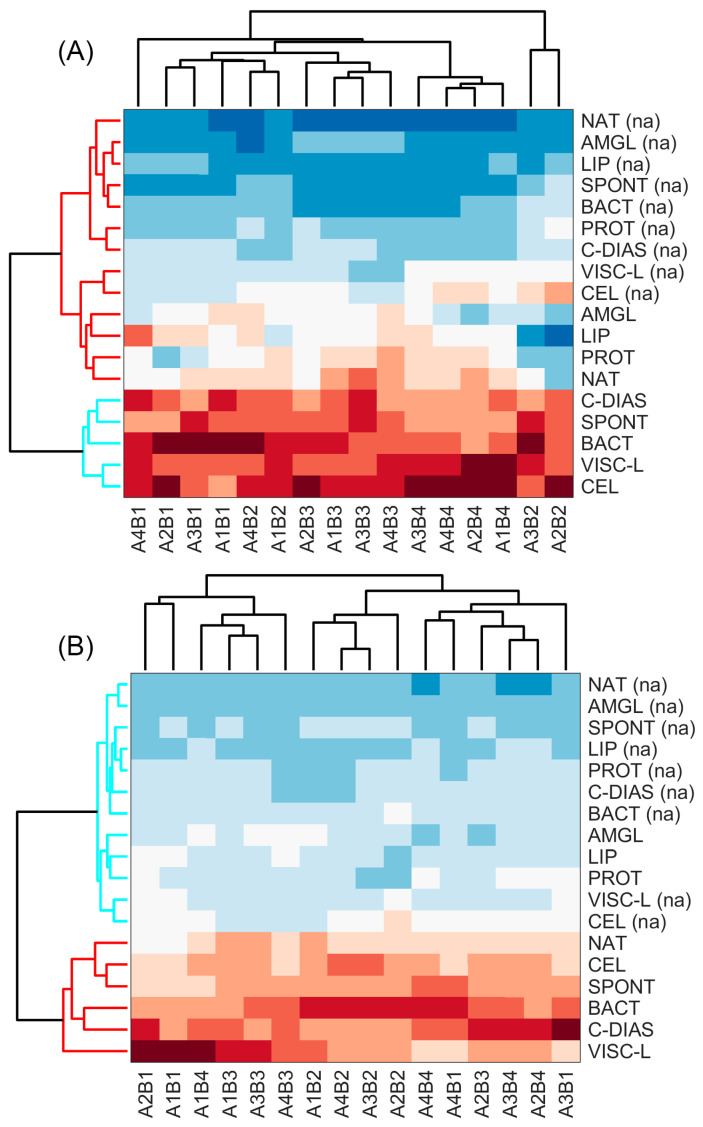
Heatmaps with dendrograms of hierarchical clustering analysis of Denmark (**A**) and Lithuanian (**B**) bee pollen samples. Bacteria: B1—*Salmonella* Typhimurium, B2—*Salmonella* Enteritidis, B3—*Listeria monocytogenes*, B4—*Staphylococcus aureus*; antibiotics: A1—ceftazidime, A2—ciprofloxacin, A3—oxytetracycline, A4—erythromycin; treatment: NAT—natural bee pollen (without treatment), SPONT—spontaneous fermentation, BACT—lactis acid fermentation, PROT—enzymatic hydrolysis by protease, LIP—hydrolysis by lipase, CEL—hydrolysis by cellulase, C-DIAS—hydrolysis by *Clara-diastase*, VISC-L—hydrolysis by *Viscozyme® L*, AMGL—hydrolysis by amyloglucosidase. Control samples are denoted as (na). The red–blue color scheme is used to visualize observed distances between samples to reveal clusters: dark blue represents the smallest, while dark red represents the largest distance values.

Firstly, this research was directed to estimation of antibacterial activity differences among tested bee pollen treatments and improvement of this characteristic after addition of antibiotics, processing each country’s data independently. The PCA was performed on the structured data matrix to extract principal orthogonal and uncorrelated components, explaining the largest portion of the data variance. The decision about number of principle components to retain was based on the Kaiser criterion and Cattell’s scree test. The analysis of the measurement results from 9 counties allowed us to reduce input data matrix dimensions from 16 variables to 3 principal components, retaining from 92.70% to 97.27% of the initial data variance. The principal components obtained were utilized as an input for hierarchical clustering analysis, employing the following HCA parameters: squared Euclidean distance served as the similarity metric, and the linkage rule was set to average. The samples from Denmark and Lithuania are presented in Figure 2, as samples from other countries performed similarly. The clustering results are presented as a heatmap with dendrogram, which was cut to form clusters at the 60% level of the maximum observed distance. Abbreviations are used in Figure 2; for example, A4B1 represents erythromycin’s (A4) impact on *Salmonella* Typhimurium (B1) after blending with specifically treated bee pollen (vertical axis).

The statistical significance of the formed clusters after HCA was tested, applying Student’s *t*-test. It was assumed that the variables of all samples falling to the same cluster were drawn from the same Gaussian distributions, and hypotheses testing about equality of the means between clusters was performed. The results revealed that observed differences between samples assigned to the distinct clusters were statistically significant at level α < 0.05. Clustering of the samples according to the antibacterial profile revealed two distinct clusters exhibiting antibacterial activity performance differences: the first “high performance antibacterial activity” group included mixtures of antibiotics and spontaneous and bacterial fermentation samples together with samples enzymatically hydrolyzed using enzymes—cellulase, *Clara-diastase,* and *Viscozyme^®^ L*. The samples containing antibiotics with natural bee pollen from Sweden, Poland, and Lithuania also were attributed to this group. The second “low performance antibacterial activity” cluster encompassed the rest of the samples, including control samples without antibiotics and mixtures of antibiotics with enzymatically hydrolyzed pollen using lipase, protease, and amyloglucosidase.

**Figure 3:**
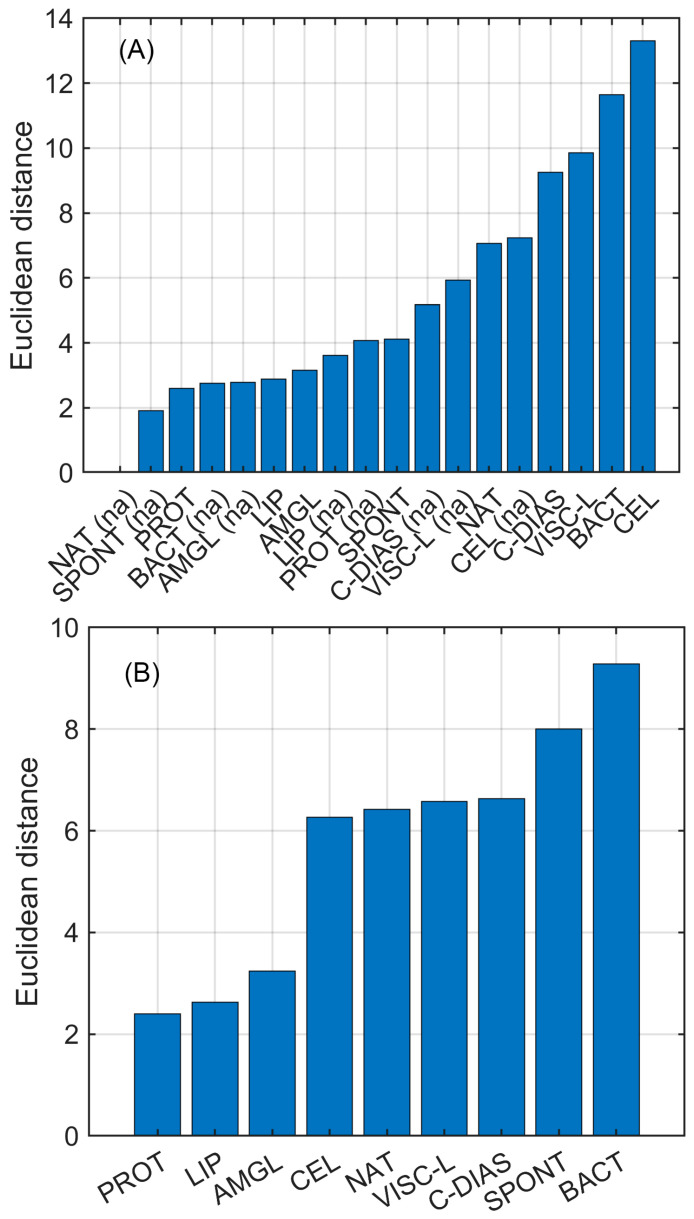
Evaluated Euclidean distance reveals antibacterial activity performance scatter among tested samples: (**A**)—distance of each tested sample from natural pollen sample; (**B**)—distance between a control fermentation sample and the same sample after addition of antibiotics (NAT—natural bee pollen (without treatment), SPONT—spontaneous fermentation, BACT—lactis acid fermentation, PROT—enzymatic hydrolysis by protease, LIP—hydrolysis by lipase, CEL—hydrolysis by cellulase, C-DIAS—hydrolysis by *Clara-diastase*, VISC-L—hydrolysis by *Viscozyme® L*, AMGL—hydrolysis by amyloglucosidase). Control samples are denoted as (na).

To expose improvements of tested pollen treatments and treated pollen mixtures with antibiotics on antibacterial activity, Euclidean distance calculation was employed. Euclidean distance can be used as a measure of similarity of objects in the N dimension space described by the same set of variables. The calculated Euclidean distance was expressed as the average value of bee pollen samples from nine countries. The sorted results in ascending order are presented in Figure 3.

Looking at Figure 3A, it is clearly seen that the best antibacterial activity performance improvement comparing to the natural bee pollen control sample is exhibited by the mixture of antibiotics and bee pollen hydrolyzed with cellulase enzyme, while the mixture of bacterial pollen fermentation with antibiotics is not far behind. The worst performances among samples mixed with antibiotics are shown by enzymatic hydrolysis with protease, lipase, and amyloglucosidase. Figure 3B shows the calculated Euclidean distance between the same bee pollen samples before (control sample) and after (mixture sample) addition of antibiotics. The bar plot reveals a non-uniform increase in antibacterial activity in the tested samples after adding antibiotics, which reaches up to 270%. These uneven changes in the antibacterial activity profile indicate a synergistic effect, which is highly demonstrated in spontaneous and bacterial fermentation. The synergism effect is weakly exposed in pollen samples enzymatically hydrolyzed with protease, lipase, and amyloglucosidase.

**Figure 4:**
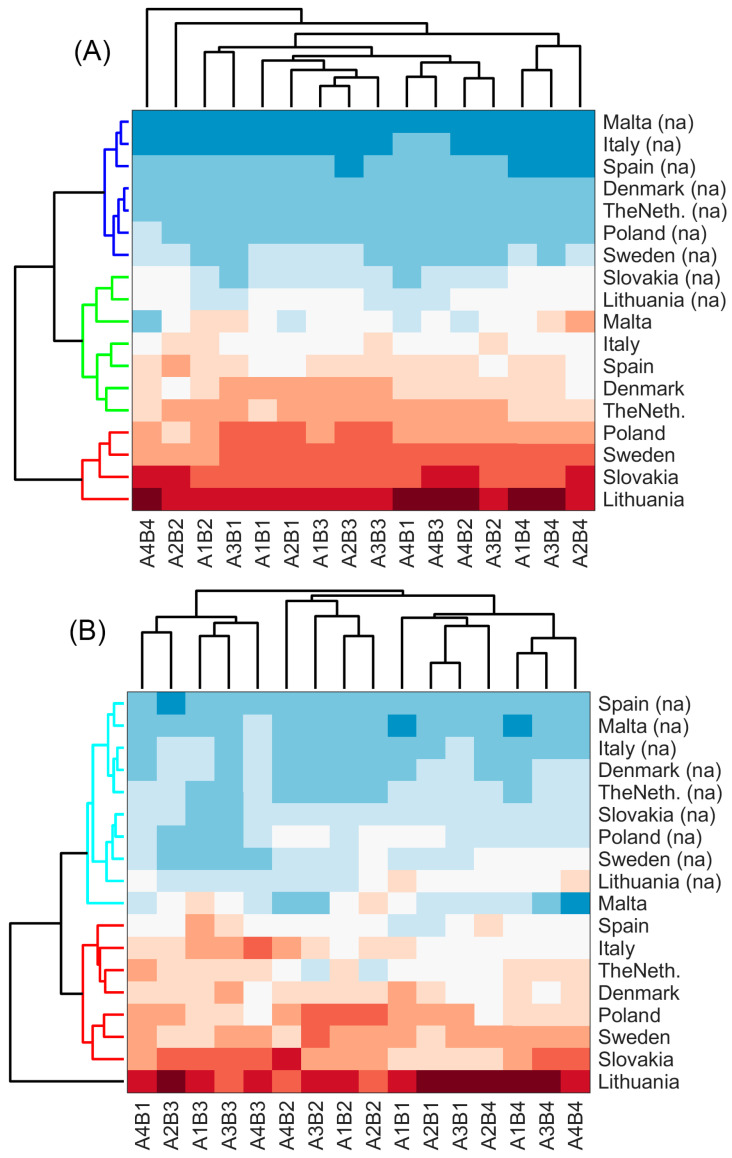
Heatmaps with dendrograms of hierarchical clustering analysis of bacterial fermentation application (**A**) and *Clara-diastase* enzyme application (**B**). Bacteria: B1—*Salmonella* Typhimurium, B2—*Salmonella* Enteritidis, B3—*Listeria monocytogenes*, B4—*Staphylococcus aureus*; antibiotics: A1—ceftazidime, A2—ciprofloxacin, A3—oxytetracycline, A4—erythromycin. Control samples are denoted as (na). The red-blue color scheme is used to visualize observed distances between samples to reveal clusters: dark blue represents the smallest, while dark red represents the largest distance values.

The successive analysis was aimed at clustering antibacterial activity data according to pollen collection region, processing each pollen treatment type independently. Following the same analysis pattern, PCA was performed and 2–4 principal components out of 16 were retained, explaining from 97.08% to 84.12% of the initial data variance. HCA with the same settings was employed to cluster the results, which are presented in Figure 4. The clustering results are presented as a heatmap with dendrogram, which was cut to form clusters at the 60% level of the maximum observed distance. Differences between samples in the formed clusters were statistically significant at α < 0.05 according to the hypotheses testing.

Figure 4A is very similar for natural pollen, bacterial and spontaneous fermentations, and also enzymatic hydrolysis using cellulase. Three clusters are suggested by the analysis according to the underlying data structure: a “high performance” cluster including Lithuanian, Polish, Slovakian and Swedish pollen, a “medium performance” cluster that included pollen samples collected in Malta, Italy, Spain, the Netherlands, and Denmark, and a “low performance” group that mainly was composed of control bee pollen samples. It is interesting to note that Lithuanian and Slovakian pollen control samples were attributed to the “medium performance” group, revealing that the antibacterial activity of these samples is very high and similar to the mixtures of southern Europe pollen samples (e.g., Malta) with antibiotics. Enzymatic hydrolysis using *Clara-diastase* and *Viscozyme^®^ L* enzymes showed another picture, presented in Figure 4B. In this case, all samples were grouped to two groups, expect the Lithuanian sample, who’s antibacterial profile was distinct from all the tests. The first cluster contained all control samples together with Malta pollen with antibiotics, while the second group included the rest of the countries’ treated pollen samples and antibiotics mixtures. The results presented in Figure 4 revealed that pollen from southern countries (Malta, Spain, and Italy) performed weaker than pollen from colder climate regions (Lithuania, Sweden, and Poland).

The clustering results of samples enzymatically hydrolyzed with lipase, protease, and amyloglucosidase did not reveal a clear clustering pattern, and the grouping was rather chaotic. Many samples were treated as separate unique clusters, which indicated that there was no expressed trend in the antibacterial activity profile and minor differences in pollen origin played a role in clustering. Clear separation of control samples and samples with antibiotics was also not observed.

**Figure 5:**
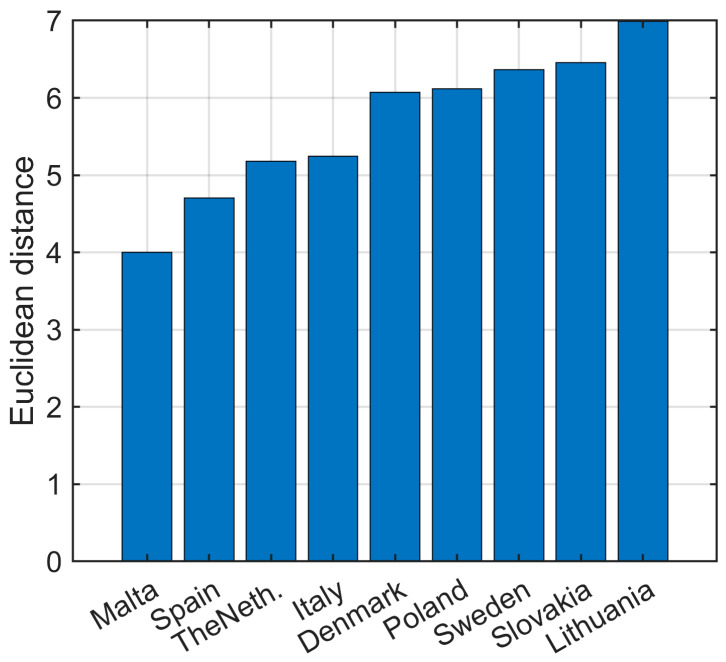
Evaluated Euclidean distance reveals antibacterial activity performance between a control country sample and the same sample after addition of antibiotics.

The Euclidean distance measure to examine pollen origin impact on antibacterial activity was calculated and expressed as the average value of nine bee pollen samples with fermentations applied. The sorted results in ascending order are presented in Figure 5, which shows the calculated Euclidean distance between bee pollen samples, collected in the same country, before (control sample) and after (mixture sample) addition of antibiotics. The bar plot affirms a non-uniform increase in antibacterial activity in the tested samples after adding antibiotics; however, the non-uniformity is not as strongly expressed as in the case of treatments.

The difference between Malta and Lithuania samples reaches 75%. Again, these uneven changes in the antibacterial activity profile indicate a synergistic effect. The synergism in the Lithuanian sample is expressed the most, while in the Malta sample it is the weakest.

The present study demonstrates the potential of various bee pollen treatment methods to enhance antibacterial activity and suggests possible synergistic effects with certain antibiotics against specific bacteria. Fermented or hydrolyzed pollen itself can be used as a natural preservative to extend the shelf life of food products or as a functional food. These preliminary findings indicate that combining bee pollen extracts with antibiotics may have potential for treating infectious diseases and addressing emerging drug resistance issues. After the selection of the most promising treatment method, this study may be expanded to include an analysis of a higher content of samples from different geographical regions and monofloral bee pollen samples, as environmental factors and local flora can significantly influence bee pollen’s composition and bioactivity. As the study was limited to a specific set of bacterial strains and antibiotics, future work should expand to a broader range of pathogens, especially antibiotic-resistant organisms, as well as a higher variety of antibacterial agents from different classes. However, further in vivo experiments are necessary to validate the efficacy of this combination in combating bacterial infections. Future research should also focus on evaluating the stability of bee pollen and antibiotic mixtures during storage, the reproducibility and optimization of these methods for industrial-scale production, and, of course, the economic feasibility of these findings.

## 3. Materials and Methods

### 3.1. Bee Pollen Samples

Nine dried bee pollen samples from various regions of Europe were collected during the flowering season, from May to August 2018, for this study. The samples included Lithuanian bee pollen from the Šiauliai region, Kuršėnai (55°59′ N 22°55′ E); Swedish bee pollen from the Hagfors region (60°02′ N 13°39′ E); Polish bee pollen from Bialystok (53°08′ N 23°08′ E); Danish bee pollen from the Alsgarde region (56°04′ N 12°32′ E); Dutch bee pollen from South Holland, Gouda (52°0′ N 4°42′ E); Slovak bee pollen from the Trnava region (48°22′ N 17°35′ E); Maltese bee pollen from the Northern region, Mellieha (35°57′ N 14°21′ E); Spanish bee pollen from the Valencia region (39°28′ N 0°22′ W); and Italian bee pollen from the Bibbiena region (43°42′ N 11°49′ E). All samples were stored at +5 °C in a dry environment, following ISO 24382:2023 [57] recommendations, for a maximum of 6 months. Prior to analysis or processing, the bee pollen samples were homogenized using a pestle and porcelain mortar.

### 3.2. Solid-State Fermentation of Bee Pollen

Solid-state fermentation of bee pollen was conducted using an inoculum of *Lacticaseibacillus rhamnosus* GG (ATCC 53103) (Gefilus, Valio Ltd., Helsinki, Finland) for bacterial fermentation and without the addition of bacteria for spontaneous fermentation. Bacterial culture viability was restored in MRS broth with Tween 80 (Biolife Italiana S.r.l., Milan, Italy) at 37 °C until reaching a cell density of 0.730 at 600 nm, determined spectrophotometrically using a Spectronic 1201 spectrophotometer (Milton Roy Co., Frederick, MD, USA).

Spontaneous and bacterial fermentations of non-pasteurized bee pollen were performed following the methodology described by Adaškevičiūtė et al. [28]. Briefly, 10 g of each bee pollen sample was moistened with 2 mL of sterile distilled water (Thermo Scientific, Fremont, CA, USA), followed by the addition of a mixture of honey and water (1.5 g honey with 2.5 mL water) after 2 h. For spontaneous fermentation, 800 µL of MRS broth with Tween 80 was added, while for bacterial fermentation, 800 µL of *L. rhamnosus* suspension (2.9 × 10^9^ colony-forming units (CFU)/mL) was added. Control samples were prepared using bidistilled water instead of bacteria or MRS broth.

The vials were placed in an incubator (Biosan, Riga, Latvia) and fermented at +37 °C for 11 and 9 days, respectively [58]. After the bioprocess, solid-state samples were extracted with 80% methanol as described in Section 3.4.

### 3.3. Enzymatic Hydrolysis of Bee Pollen

Enzymatic hydrolysis of bee pollen was conducted using six commercial enzymes: two enzyme mixtures—*Clara-diastase* and *Viscozyme^®^ L*—and four pure enzymes—lipase from *Aspergillus oryzae*, protease from *Bacillus* species, amyloglucosidase from *A. niger*, and cellulase from *A. niger* (Sigma-Aldrich Corporation, Taufkirchen, Germany). The bioprocess followed the methodology of our previous study [31]. Briefly, 1 g of bee pollen was mixed with 0.5 mL of sterile distilled water and treated at 121 °C for 15 min. The resulting mixture was supplemented with the optimal amount of each enzyme (175 µL of cellulase, *Viscozyme^®^ L*, and *Clara-diastase*; 200 µL of protease, lipase, and amyloglucosidase) and incubated for the optimal duration (3 h 15 min for cellulase, *Viscozyme^®^ L*, and *Clara-diastase*; 3 h 45 min for protease, lipase, and amyloglucosidase) [31]. Control samples were prepared using the corresponding buffer instead of the tested enzyme.

Vials containing the samples were boiled for 2 min to terminate the bioprocess, and the resulting mixtures were extracted using 80% methanol as described in Section 3.4.

### 3.4. Extraction of Bee Pollen Samples

Natural bee pollen samples were moistened with bidistilled water in order to achieve the appropriate consistency. All samples (natural, after fermentation or enzymatic hydrolysis) were extracted in a 1:10 ratio with 80% methanol (Sigma-Aldrich Corporation, Taufkirchen, Germany) [29]. After extraction, samples were filtered through 7–10 μm paper filter (Labbox, Barcelona, Spain) and 0.22 μm polyvinylidene fluoride (PVDF) membrane filter (BGB Analytik, Alexandria, VA, USA).

### 3.5. Antibacterial Activity and Interaction with Antibiotics Evaluation

The antibacterial activity of bee pollen extracts, antibiotics, or bee pollen and antibiotic mixtures was evaluated using the agar well diffusion method [29]. The inhibition of growth of four pathogenic bacteria was tested, including Gram-positive *Staphylococcus aureus* ATCC 6538 (LGC Standards, Lomianki, Poland) and *Listeria monocytogenes* ATCC 7644, as well as Gram-negative *Salmonella enterica* subsp. *enterica* serovar Enteritidis ATCC 13076 and *Salmonella enterica* subsp. *enterica* serovar Typhimurium ATCC 14028. Revitalization of indicator strains was performed using LB medium (Carl Roth, Karlsruhe, Germany). Succinctly, an overnight-grown bacterial inoculum in sterile LB-agar medium was spread onto Petri dishes. The following antibiotics were used during the analysis: ceftazidime pentahydrate (98%), ciprofloxacin (98%), oxytetracycline dihydrate (95%) (Acros Organics, Geel, Belgium), and erythromycin (≥93%) (Carl Roth Gmbh & Co. Kg, Karlsruhe, Germany).

Before the antibacterial test, three series of samples were prepared: (1) bee pollen extract were diluted with sterile water (1:1); (2) 30 µg/mL aqueous antibiotic solutions were diluted with sterile water (1:1); and (3) bee pollen extract mixed with 30 µg/mL corresponding antibiotic in a ratio (1:1). The prepared solutions were tested for antibacterial activity as described below.

Three wells were cut in each dish, and a drop of liquid agar was used at the bottom to prevent extract leakage. Wells were filled with 100 µL of 0.9% NaCl, 80% methanol, or testing solution. Ten repetitions were performed for each sample (bee pollen, antibiotics, or their mixture).

Antibacterial activity was evaluated by inhibition zone expressed by millimeters after incubation for 24 h at +37 °C. In vitro interactions (*I_combination_*) between bee pollen extracts and antibacterial agents were determined and evaluated using the subsequent formula:

*I_combination_* = *A_e_* + *A_a_*, (1)

where *A_e_*—inhibition zone (mm) of bee pollen extract, *A_a_*—inhibition zone (mm) of antibiotics. The results were interpreted as follows:

- Synergistic, when the combined antibacterial activity was greater than the sum of the bee pollen extract and antibiotic antibacterial activity.
- Additive, when the combined antibacterial activity was equal to the sum of the bee pollen extract and antibiotic antibacterial activity.
- Antagonistic, when the combined antibacterial activity was weaker than the sum of the bee pollen extract and antibiotic antibacterial activity.

### 3.6. Statistical Analysis

The data were systematized using MS Excel 15.11.2 (2015, Microsoft, Redmond, WA, USA) software, and the results were subjected to analysis through linear regression modeling. Subsequently, chemometric analysis was carried out using MATLAB v23.2.0—R2023b Update 6 (2023, MathWorks, Natick, MA, USA) software.

Measured data were organized into a multidimensional matrix, representing 9 countries (Denmark, Sweden, Poland, Lithuania, Slovakia, the Netherlands, Italy, Spain, and Malta), 4 bacteria (*Salmonella* Typhimurium (B1), *Salmonella* Enteritidis (B2), *Listeria monocytogenes* (B3), and *Staphylococcus aureus* (B4)), 4 antibiotics (ceftazidime (A1), ciprofloxacin (A2), oxytetracycline (A3), and erythromycin (A4)), and 9 treatments (natural bee pollen (NAT), spontaneous (SPONT) and bacterial (BACT) fermentation, and enzymatic hydrolysis using enzymes—protease (PROT), lipase (LIP), cellulase (CEL), *Clara-diastase* (C-DIAS), *Viscozyme^®^ L* (VISC-L), amyloglucosidase (AMGL)) conducting 10 repeated inhibition zone measurements. The dataset also included the results of 10 measurements of control samples for each fermentation and enzymatic hydrolysis, conducted without antibiotics. The final size of the data matrix prepared for chemometric analysis was 9 × 4 × 5 × 90. The data were analyzed from the perspective of pollen treatment (fermentation, enzymatic hydrolysis) and pollen origin (country) to determine treatment ways and pollen collection regions contributing the most to the antibiotic activity, emphasizing the synergistic effect.

Data preprocessing involved standardizing the variables by subtracting the mean and then dividing by the standard deviation. ANOVA was used for hypothesis testing to identify statistically significant changes in antibacterial activity values after fermentation or enzymatic hydrolysis, as well as following the addition of antibiotics, with a significance level of *p* ≤ 0.05.

Clustering analysis involved a data standardization procedure, principal component analysis (PCA), hierarchical clustering analysis (HCA), and Euclidean distance between samples estimation. PCA was aimed at removing correlation in the initial variables and reducing the number of data dimensions. Applied HCA allowed us to group tested samples to clusters according to similarities among them according to the antibacterial activity profile. For statistical power improvement, hypotheses testing was employed to test statistical significance of the clusters formed by HCA. Student’s t statistic was used to test statistically significant differences between variables, describing samples assigned to distinct clusters.

The statistical analysis was aimed to reveal optimal treatment ways and pollen collection regions in Europe, exposing better antibacterial performance and emphasizing the effect of added antibiotics. Euclidean distance calculation was employed to investigate antibacterial activity improvements between bee pollen samples before and after addition of antibiotics and evaluate the synergistic effect. The *ED* value was calculated in an orthogonal variable space after PCA (i.e., principal component space) using the following equation:

(2) $$ED=\sqrt{\sum_{i=1}^{N}{\left(S_{control\_i}-S_{i}\right)}^{2}}$$

where *S_control_* denotes a control pollen sample, *S* denotes a sample, for which Euclidean distance is being evaluated, *N* is the number of variables, describing the samples.

## 4. Conclusions

Solid-state fermentation of bee pollen positively impacts antibacterial activity. Spontaneous fermentation significantly (*p* ≤ 0.05) increased antibacterial activity by 1.04–2.92 times, while bacterial fermentation increased it by 1.18–4.08 times across all tested bee pollen samples. Additionally, interactions of natural and treated bee pollen extracts in combination with antibiotics were studied. Among all combinations tested against four Gram-positive and Gram-negative pathogenic bacteria, bee pollen extracts combined with four different antimicrobial agents exhibited 80.79% synergistic, 18.75% antagonistic, and 0.46% additive properties. These findings suggest that bee pollen extracts contain natural inhibitors operating through various mechanisms, potentially enhancing the effectiveness of antibiotics.

Chemometric analysis revealed that the highest antibacterial activity was achieved through bacterial fermentation and enzymatic hydrolysis with cellulolytic enzymes (cellulase, *Clara-diastase,* and *Viscozyme^®^ L*), while the weakest activity was observed in samples enzymatically hydrolyzed with lipase, protease, and amyloglucosidase. The most pronounced synergistic effects after adding antibiotics were observed in samples from spontaneous and bacterial fermentation. Pollen from southern countries (Malta, Spain, and Italy) exhibited weaker antibacterial activity and less interaction with antibiotics compared to pollen from colder regions (Lithuania, Sweden, and Poland).

## Figures and Tables

**Table 1:** An impact of fermentation and enzymatic hydrolysis on antibacterial activity of tested bee pollen and interaction with antibiotics (n = 10; SY—synergistic effect, AN—antagonistic effect; *i*—observed significant changes after effect with antibiotics are labeled as “*i*” (increased) when *p* ≤ 0.05).

Treatment	Used Antibiotic	Against *Salmonella* Typhimurium				Against *Salmonella* Enteritidis				Against *L. monocytogenes*				Against *S. aureus*			
		Inhibition Zone, mm	MIN–MAX, mm	SY	AN	Inhibition Zone, mm	MIN–MAX, mm	SY	AN	Inhibition Zone, mm	MIN–MAX, mm	SY	AN	Inhibition Zone, mm	MIN–MAX, mm	SY	AN
Natural	Without antibiotic	4.00 ± 2.98	0.91–9.14	−	−	7.79 ± 4.09	2.13–15.31	−	−	7.68 ± 2.71	5.10–12.90	−	−	8.09 ± 4.27	2.21–15.42	−	−
	Ceftazidime	11.76 ± 5.32 *^i^*	5.15–20.80	9	0	20.61 ± 9.19 *^i^*	9.70–36.00	9	0	19.43 ± 4.10 *^i^*	13.60–26.65	9	0	20.59 ± 7.84 *^i^*	8.35–32.85	9	0
	Ciprofloxacin	11.38 ± 4.12 *^i^*	6.30–17.40	9	0	15.04 ± 6.10 *^i^*	7.95–24.25	7	2	13.87 ± 4.14 *^i^*	7.05–20.00	8	1	20.25 ± 5.95 *^i^*	11.80–30.60	9	0
	Oxytetracycline	15.37 ± 5.84 *^i^*	7.90–22.60	9	0	19.63 ± 8.70 *^i^*	8.85–33.10	9	0	22.28 ± 6.35 *^i^*	11.90–31.15	9	0	22.73 ± 7.18 *^i^*	10.35–31.25	9	0
	Erythromycin	16.64 ± 6.76 *^i^*	8.85–25.90	9	0	20.11 ± 8.49 *^i^*	8.55–33.45	9	0	24.16 ± 7.94 *^i^*	11.00–37.50	9	0	20.25 ± 7.93 *^i^*	4.30–32.00	8	1
Spontaneous fermentation	Without antibiotic	5.16 ± 2.98	1.51–10.56	−	−	10.95 ± 3.82	5.49–18.95	−	−	9.31 ± 2.91	6.00–14.27	−	−	11.28 ± 3.83	5.59–19.05	−	−
	Ceftazidime	17.27 ± 5.24 *^i^*	9.80–27.05	9	0	30.91 ± 5.20 *^i^*	22.55–38.30	9	0	22.50 ± 4.47 *^i^*	15.85–29.30	9	0	23.56 ± 6.63 *^i^*	15.85–35.20	9	0
	Ciprofloxacin	16.12 ± 4.21 *^i^*	8.95–22.65	9	0	21.69 ± 5.80 *^i^*	12.95–29.80	9	0	20.57 ± 3.48 *^i^*	14.25–25.15	9	0	23.87 ± 6.71 *^i^*	14.55–34.70	9	0
	Oxytetracycline	21.37 ± 5.35 *^i^*	13.55–28.20	9	0	27.35 ± 7.43 *^i^*	14.90–37.40	9	0	25.87 ± 6.32 *^i^*	16.30–35.65	9	0	25.89 ± 7.39 *^i^*	14.60–37.80	9	0
	Erythromycin	23.47 ± 8.76 *^i^*	9.30–37.85	9	0	26.09 ± 6.40 *^i^*	16.25–36.50	9	0	30.29 ± 8.85 *^i^*	18.05–39.65	9	0	23.63 ± 9.35 *^i^*	6.65–39.10	8	1
Bacterial fermentation	Without antibiotic	6.06 ± 3.17	1.62–11.94	−	−	12.84 ± 4.81	7.07–21.54	−	−	10.23 ± 2.96	6.54–15.58	−	−	13.16 ± 4.85	7.12–21.62	−	−
	Ceftazidime	20.90 ± 6.50 *^i^*	12.50–30.50	9	0	36.44 ± 7.34 *^i^*	29.10–45.25	9	0	23.61 ± 4.47 *^i^*	17.55–30.35	9	0	26.64 ± 6.20 *^i^*	18.95–38.05	9	0
	Ciprofloxacin	21.15 ± 6.57 *^i^*	10.95–29.35	9	0	27.08 ± 5.30 *^i^*	20.45–35.10	9	0	22.12 ± 3.95 *^i^*	15.40–27.60	9	0	26.67 ± 6.42 *^i^*	18.50–36.70	9	0
	Oxytetracycline	26.95 ± 6.18 *^i^*	17.15–36.70	9	0	31.71 ± 7.94 *^i^*	20.10–43.75	9	0	26.81 ± 6.06 *^i^*	16.40–35.95	9	0	29.68 ± 6.52 *^i^*	21.40–41.55	9	0
	Erythromycin	29.13 ± 11.09 *^i^*	10.90–41.85	9	0	32.16 ± 9.33 *^i^*	18.90–41.30	9	0	32.67 ± 9.47 *^i^*	20.65–44.20	9	0	26.09 ± 9.77 *^i^*	9.90–43.70	8	1
Enzymatic hydrolysis with protease	Without antibiotic	7.77 ± 2.51	3.50–11.45	−	−	10.72 ± 3.34	7.25–16.60	−	−	11.37 ± 1.56	9.55–14.80	−	−	13.64 ± 3.98	9.15–21.70	−	−
	Ceftazidime	8.27 ± 3.15	3.95–14.10	2	7	12.80 ± 5.18	5.65–20.45	2	7	13.54 ± 2.99	6.80–16.65	4	4	16.05 ± 4.93	8.10–22.55	3	6
	Ciprofloxacin	7.84 ± 4.02	2.65–15.95	1	7	9.77 ± 4.45	2.40–16.15	1	8	11.63 ± 3.14	6.25–15.75	1	8	15.41 ± 5.85	9.00–27.00	5	4
	Oxytetracycline	9.87 ± 4.43	3.15–19.50	1	8	12.16 ± 3.70	5.55–16.40	3	6	15.62 ± 3.81	8.05–20.00	6	3	17.21 ± 6.14	6.95–26.95	5	4
	Erythromycin	11.30 ± 4.36	3.95–18.60	4	5	11.89 ± 4.55	3.10–17.30	2	6	19.92 ± 6.26	11.20–32.90	8	1	14.97 ± 6.60	2.90–24.50	2	7
Enzymatic hydrolysis with lipase	Without antibiotic	6.48 ± 2.16	3.35–9.75	−	−	9.12 ± 3.42	5.30–15.95	−	−	10.42 ± 1.58	9.00–13.85	−	−	12.47 ± 4.52	5.95–21.05	−	−
	Ceftazidime	7.86 ± 5.20	2.50–18.45	3	6	11.92 ± 5.69	5.60–21.95	2	7	11.98 ± 2.83	8.85–16.50	2	7	15.14 ± 6.26	3.90–23.50	5	4
	Ciprofloxacin	9.18 ± 4.20	2.55–15.95	3	5	9.47 ± 3.45	5.45–15.45	3	6	11.37 ± 3.01	6.40–15.90	1	8	15.05 ± 5.64	5.70–23.05	5	4
	Oxytetracycline	10.19 ± 4.53	3.90–15.55	5	4	12.09 ± 6.73	5.50–25.05	2	7	14.96 ± 4.01	8.40–19.65	6	3	17.75 ± 7.33	7.30–28.20	6	3
	Erythromycin	13.23 ± 7.48	3.50–24.95	6	3	14.44 ± 6.40	5.40–25.65	6	3	17.35 ± 5.44	9.80–26.85	7	2	14.42 ± 6.18	4.45–22.55	2	7
Enzymatic hydrolysis with cellulase	Without antibiotic	11.40 ± 4.19	5.75–18.50	−	−	17.86 ± 4.26	12.20–23.35	−	−	16.74 ± 4.02	13.55–26.65	−	−	20.68 ± 5.56	13.50–26.85	−	−
	Ceftazidime	19.64 ± 4.96 *^i^*	12.45–26.90	9	0	32.54 ± 6.04 *^i^*	23.15–42.00	9	0	25.77 ± 2.52 *^i^*	22.60–30.35	8	1	30.19 ± 7.21 *^i^*	19.00–38.45	9	0
	Ciprofloxacin	19.29 ± 4.47 *^i^*	11.90–23.90	9	0	24.66 ± 3.74 *^i^*	19.45–29.15	9	0	24.73 ± 1.75 *^i^*	22.05–27.65	8	1	29.01 ± 6.07 *^i^*	18.45–36.95	8	1
	Oxytetracycline	22.21 ± 4.40 *^i^*	16.00–29.55	9	0	29.02 ± 7.17 *^i^*	20.10–41.65	9	0	29.06 ± 3.50 *^i^*	25.35–36.00	9	0	32.73 ± 7.16 *^i^*	21.35–41.50	9	0
	Erythromycin	26.89 ± 7.46 *^i^*	18.00–38.85	9	0	30.75 ± 8.74 *^i^*	18.95–42.90	9	0	34.91 ± 8.26 *^i^*	27.90–42.25	9	0	30.55 ± 9.45 *^i^*	9.85–41.70	8	1
Enzymatic hydrolysis with *Clara-diastase*	Without antibiotic	9.39 ± 3.57	4.25–15.45	−	−	13.79 ± 3.24	10.60–18.60	−	−	12.46 ± 1.37	9.40–14.50	−	−	15.38 ± 4.34	9.75–22.20	−	−
	Ceftazidime	17.87 ± 5.83 *^i^*	9.00–28.40	8	1	29.41 ± 6.26 *^i^*	22.55–40.10	9	0	26.25 ± 5.12 *^i^*	22.15–36.25	9	0	25.54 ± 7.40 *^i^*	14.25–41.05	9	0
	Ciprofloxacin	18.81 ± 8.51 *^i^*	11.60–39.85	9	0	21.57 ± 3.75 *^i^*	15.95–28.00	9	0	21.09 ± 4.05 *^i^*	16.05–29.80	9	0	25.36 ± 7.90 *^i^*	17.30–42.80	9	0
	Oxytetracycline	22.10 ± 11.44 *^i^*	10.75–43.10	9	0	25.39 ± 9.06 *^i^*	11.90–37.35	8	1	26.86 ± 3.98 *^i^*	20.45–32.35	9	0	27.09 ± 9.27 *^i^*	13.00–41.55	9	0
	Erythromycin	25.41 ± 8.81 *^i^*	10.80–41.95	9	0	26.91 ± 9.33 *^i^*	11.50–41.30	8	1	33.00 ± 9.86 *^i^*	17.65–44.85	9	0	24.07 ± 8.98 *^i^*	5.90–37.30	8	1
Enzymatic hydrolysis with *Viscozyme^®^ L*	Without antibiotic	10.16 ± 4.03	4.90–17.50	−	−	15.48 ± 4.15	9.75–21.30	−	−	14.12 ± 1.47	12.30–15.95	−	−	17.32 ± 4.85	11.35–25.65	−	−
	Ceftazidime	21.24 ± 11.73 *^i^*	9.75–45.40	9	0	32.38 ± 6.41 *^i^*	25.55–43.45	9	0	25.63 ± 5.26 *^i^*	20.70–37.85	9	0	29.62 ± 9.34 *^i^*	15.80–45.90	9	0
	Ciprofloxacin	19.76 ± 10.11 *^i^*	8.95–43.60	9	0	22.01 ± 4.20 *^i^*	14.50–28.25	9	0	22.36 ± 2.17 *^i^*	19.20–25.10	9	0	26.38 ± 6.88 *^i^*	14.45–35.50	9	0
	Oxytetracycline	20.79 ± 5.44 *^i^*	13.70–28.25	9	0	28.70 ± 6.79 *^i^*	17.45–37.40	9	0	28.77 ± 10.68 *^i^*	21.25–40.80	9	0	28.86 ± 9.15 *^i^*	14.60–39.90	9	0
	Erythromycin	23.09 ± 8.50 *^i^*	9.35–37.10	9	0	27.27 ± 6.48 *^i^*	19.50–36.55	9	0	35.47 ± 9.64 *^i^*	23.05–44.50	9	0	26.33 ± 9.73 *^i^*	6.75–39.05	8	1
Enzymatic hydrolysis with amylogluco-sidase	Without antibiotic	5.29 ± 2.83	0.95–9.55	−	−	8.64 ± 3.80	4.70–16.00	−	−	9.77 ± 1.92	7.65–13.50	−	−	10.89 ± 4.01	5.35–17.95	−	−
	Ceftazidime	7.44 ± 3.30	1.85–12.40	2	7	13.48 ± 5.89	7.10–25.65	6	3	12.69 ± 3.67	7.30–17.45	5	4	14.89 ± 6.30	6.85–25.50	7	2
	Ciprofloxacin	8.05 ± 3.43	2.65–12.95	4	5	9.14 ± 4.02	3.60–16.95	1	8	9.92 ± 2.75	6.45–13.50	0	8	12.77 ± 6.30	4.80–24.10	3	6
	Oxytetracycline	11.84 ± 7.40	4.35–28.35	6	2	12.47 ± 5.05	5.55–20.20	4	5	15.93 ± 3.34	9.05–19.90	8	1	17.98 ± 7.22	5.25–28.05	8	1
	Erythromycin	12.48 ± 4.35	6.35–18.70	9	0	14.12 ± 5.83	6.40–21.80	6	3	21.46 ± 5.29	10.25–28.40	8	1	16.20 ± 5.53	3.55–21.30	7	2

## Data Availability

Data will be made available on request.

## Supplementary Materials

The following supporting information can be downloaded at: https://www.mdpi.com/article/10.3390/ph18010015/s1, Figure S1: Tested bee pollen samples from various European countries; Table S1: An impact of fermentation and enzymatic hydrolysis on antibacterial activity of tested bee pollen and interaction with antibiotics.
