# Supplementary material for: Improved Antibacterial Properties of Fermented and Enzymatically Hydrolyzed Bee Pollen and Its Combined Effect with Antibiotics
Source: Pharmaceuticals (Basel). 2024 Dec 26;18(1):15. doi: 10.3390/ph18010015 (PMC11768341; doi:10.3390/ph18010015)
Supplement: Supplementary file 1 [file pharmaceuticals-18-00015-s001.zip › pharmaceuticals-3349633-supplementary.pdf]

Improved Antibacterial Properties of Fermented and Enzymatically Hydrolyzed Bee Pollen and Its Combined Effect with Antibiotics

Vaida Damulienė [1], Vilma Kaškonienė [1,*], Paulius Kaškonas [2] , Rūta Mickienė [1] and Audrius Maruška [1]

[1] Instrumental Analysis Open Access Centre, Vytautas Magnus University, LT-44404 Kaunas, Lithuania.
[2] Institute of Metrology, Kaunas University of Technology, LT-51368 Kaunas, Lithuania.
* Correspondence: vilma.kaskoniene@vdu.lt (V.K.)

Supplementary material

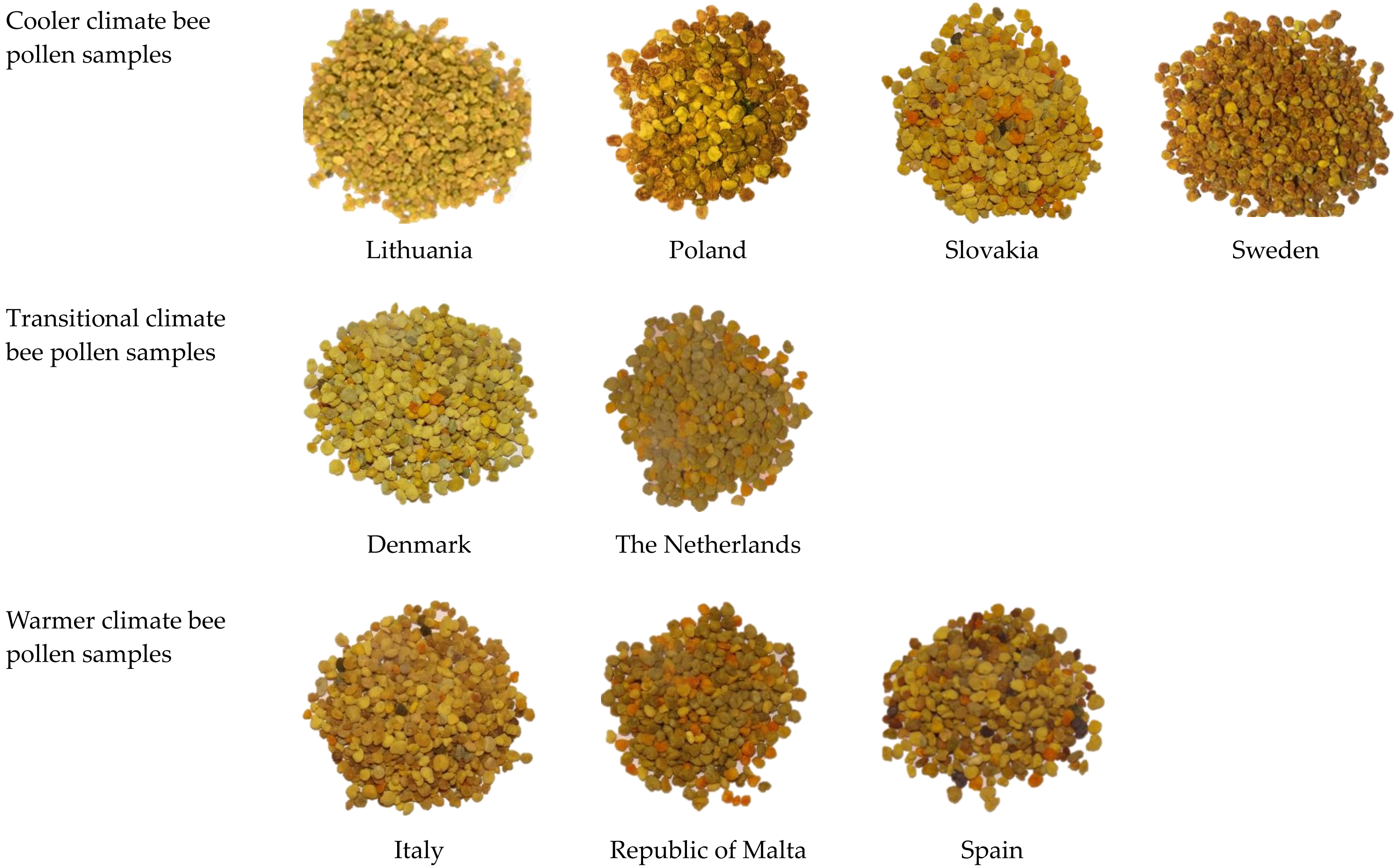

Figure S1. Tested bee pollen samples from various European countries

Table S1

An impact of fermentation and enzymatic hydrolysis on antibacterial activity of tested bee pollen and interaction with antibiotics (n = 10; antibacterial activity expressed by inhibition zone in mm)

Treatment	Usage of antibiotic[1]	Bee pollen sample								
		Denmark	Sweden	Poland	Lithuania	Slovakia	Netherlands	Italy	Spain	Malta
		**Antibacterial activity against *Staphylococcus aureus***								
Control	Ceftazidime	2.46 ± 0.20								
	Ciprofloxacin	2.57 ± 0.12								
	Oxytetracycline	1.93 ± 0.07								
	Erythromycin	3.52 ± 0.25								
Natural	n/a	8.28 ± 0.25	12.14 ± 0.49	8.40 ± 0.53	15.42 ± 0.14	11.03 ± 0.50	7.13 ± 0.40	3.75 ± 0.30	4.52 ± 0.44	2.21 ± 0.07
	Ceftazidime	20.70 ± 0.79	27.20 ± 2.07	23.75 ± 0.54	32.85 ± 0.53	26.65 ± 0.97	17.20 ± 1.01	12.00 ± 0.91	16.60 ± 1.39	8.35 ± 0.78
	Ciprofloxacin	20.30 ± 0.79	24.85 ± 0.88	20.80 ± 0.89	30.60 ± 0.84	25.45 ± 0.93	14.80 ± 1.32	15.60 ± 1.26	18.05 ± 1.23	11.80 ± 0.63
	Oxytetracycline	19.95 ± 0.16	29.45 ± 1.59	25.10 ± 1.45	31.25 ± 1.36	30.85 ± 1.20	23.10 ± 0.57	16.30 ± 1.18	18.25 ± 0.54	10.35 ± 1.42
	Erythromycin	21.10 ± 1.13	24.15 ± 1.60	20.40 ± 1.54	32.00 ± 0.82	26.60 ± 0.66	21.95 ± 1.34	13.25 ± 1.32	18.50 ± 1.51	4.30 ± 1.21
Spontaneous fermentation	n/a	10.76 ± 0.10	12.65 ± 0.46	11.78 ± 0.49	19.05 ± 0.55	13.34 ± 0.18	11.78 ± 0.44	8.18 ± 0.19	8.42 ± 0.47	5.59 ± 0.58
	Ceftazidime	22.05 ± 0.60	28.30 ± 1.25	24.30 ± 0.67	35.20 ± 0.54	30.60 ± 0.39	20.10 ± 0.57	16.40 ± 0.46	19.20 ± 0.59	15.85 ± 1.20
	Ciprofloxacin	21.00 ± 0.41	29.60 ± 1.29	25.60 ± 0.84	34.70 ± 1.25	30.75 ± 0.26	21.40 ± 1.17	17.40 ± 0.21	19.85 ± 0.71	14.55 ± 0.64
	Oxytetracycline	22.85 ± 0.53	31.95 ± 1.12	28.60 ± 1.35	37.80 ± 1.80	32.15 ± 0.34	25.50 ± 1.00	19.10 ± 0.74	20.50 ± 0.78	14.60 ± 0.39
	Erythromycin	23.55 ± 0.64	26.60 ± 2.13	23.55 ± 1.01	39.10 ± 0.81	33.45 ± 1.09	23.60 ± 0.97	16.40 ± 1.20	19.80 ± 1.40	6.65 ± 0.24
Bacterial fermentation	n/a	11.78 ± 0.66	14.36 ± 0.49	13.34 ± 0.27	21.62 ± 0.33	19.56 ± 1.10	12.83 ± 0.22	8.67 ± 0.71	9.18 ± 0.14	7.12 ± 0.54
	Ceftazidime	24.95 ± 0.37	32.05 ± 0.72	26.05 ± 0.76	38.05 ± 1.09	31.40 ± 2.08	25.60 ± 0.52	18.95 ± 1.23	23.25 ± 0.35	19.50 ± 1.33
	Ciprofloxacin	21.45 ± 0.69	30.70 ± 1.49	26.85 ± 1.70	36.70 ± 0.79	34.95 ± 1.69	22.80 ± 1.40	18.50 ± 1.15	20.75 ± 0.54	27.30 ± 1.23
	Oxytetracycline	26.50 ± 0.78	34.75 ± 1.44	31.10 ± 0.81	41.55 ± 1.04	35.35 ± 0.34	27.60 ± 0.88	21.40 ± 0.32	23.70 ± 0.63	25.15 ± 1.00
	Erythromycin	24.15 ± 0.58	29.30 ± 1.27	26.35 ± 1.53	43.70 ± 0.86	35.75 ± 0.49	25.45 ± 0.80	17.70 ± 0.75	22.50 ± 0.33	9.90 ± 0.66
Enzymatic hydrolysis with protease	n/a	12.90 ± 1.29	15.05 ± 1.21	14.00 ± 0.88	21.70 ± 0.92	16.75 ± 0.68	13.45 ± 0.86	10.45 ± 0.86	9.15 ± 0.53	9.30 ± 0.86
	Ceftazidime	18.85 ± 0.58	16.15 ± 0.47	22.05 ± 0.37	22.55 ± 0.28	17.00 ± 0.02	15.15 ± 0.41	9.50 ± 0.02	15.10 ± 0.21	8.10 ± 0.32
	Ciprofloxacin	18.15 ± 0.24	14.70 ± 1.03	9.50 ± 0.24	27.00 ± 0.02	20.75 ± 0.59	10.30 ± 0.67	14.50 ± 0.41	14.75 ± 0.35	9.00 ± 0.02
	Oxytetracycline	21.80 ± 1.14	15.65 ± 0.75	13.15 ± 0.34	26.95 ± 0.16	21.00 ± 1.20	19.20 ± 1.53	10.95 ± 0.69	19.25 ± 0.26	6.95 ± 0.16
	Erythromycin	20.25 ± 0.86	15.30 ± 0.54	11.05 ± 0.16	24.50 ± 0.24	19.05 ± 1.30	16.40 ± 0.81	8.25 ± 0.68	17.05 ± 0.16	2.90 ± 0.32
	n/a	11.65 ± 1.25	14.50 ± 1.05	13.40 ± 0.74	21.05 ± 0.86	16.10 ± 0.88	12.15 ± 1.06	5.95 ± 0.83	8.80 ± 1.34	8.65 ± 0.58
	Ceftazidime	17.50 ± 0.02	23.50 ± 1.27	12.10 ± 0.21	22.55 ± 0.16	19.75 ± 1.18	13.00 ± 0.85	10.95 ± 0.50	13.05 ± 0.69	3.90 ± 0.39

Treatment	Usage of antibiotic[1]	Bee pollen sample								
		**Denmark**	**Sweden**	**Poland**	**Lithuania**	**Slovakia**	**Netherlands**	**Italy**	**Spain**	**Malta**
Enzymatic hydrolysis with lipase	Ciprofloxacin	16.45 ± 0.16	20.80 ± 1.30	13.40 ± 0.32	23.05 ± 0.28	20.65 ± 0.63	12.80 ± 0.54	10.85 ± 0.34	11.75 ± 0.35	5.70 ± 0.26
	Oxytetracycline	19.70 ± 0.89	28.20 ± 0.67	11.35 ± 0.24	25.00 ± 0.24	21.90 ± 1.43	22.50 ± 0.88	7.30 ± 1.74	13.60 ± 0.52	10.20 ± 0.35
	Erythromycin	18.35 ± 0.85	22.55 ± 0.44	13.85 ± 0.34	21.00 ± 0.02	18.85 ± 1.43	12.50 ± 0.41	8.45 ± 2.65	9.75 ± 1.93	4.45 ± 0.16
Enzymatic hydrolysis with cellulase	n/a	19.15 ± 0.85	26.80 ± 0.82	23.10 ± 0.88	26.75 ± 0.95	26.85 ± 1.18	20.80 ± 0.63	15.00 ± 1.18	13.50 ± 1.33	14.15 ± 0.82
	Ceftazidime	30.55 ± 1.40	38.05 ± 0.55	31.55 ± 2.28	38.45 ± 1.50	36.55 ± 1.28	29.90 ± 0.57	28.20 ± 0.89	19.00 ± 1.13	19.50 ± 0.41
	Ciprofloxacin	27.90 ± 1.20	36.70 ± 1.36	34.90 ± 1.22	36.95 ± 0.16	26.75 ± 1.70	26.45 ± 1.01	25.75 ± 1.86	18.45 ± 0.72	27.20 ± 1.38
	Oxytetracycline	32.65 ± 1.47	41.50 ± 1.72	35.30 ± 0.89	34.70 ± 0.42	41.50 ± 0.58	31.55 ± 0.93	28.65 ± 0.24	21.35 ± 0.78	25.20 ± 1.32
	Erythromycin	30.45 ± 1.69	41.70 ± 0.75	35.70 ± 1.49	35.00 ± 1.15	36.85 ± 0.58	29.25 ± 1.51	27.60 ± 0.94	26.55 ± 0.44	9.85 ± 1.29
Enzymatic hydrolysis with *Clara-diastase*	n/a	13.70 ± 0.67	21.05 ± 0.80	17.00 ± 0.71	22.20 ± 0.75	17.45 ± 0.60	14.10 ± 1.13	11.65 ± 0.94	11.50 ± 1.13	9.75 ± 1.09
	Ceftazidime	25.50 ± 1.49	27.80 ± 1.14	25.10 ± 0.39	41.05 ± 0.37	30.25 ± 1.21	23.65 ± 3.52	20.70 ± 0.59	21.55 ± 1.01	14.25 ± 0.26
	Ciprofloxacin	22.15 ± 0.78	30.70 ± 0.98	22.10 ± 0.77	43.80 ± 0.35	26.45 ± 0.28	21.40 ± 0.32	20.30 ± 0.26	24.00 ± 0.82	17.30 ± 0.63
	Oxytetracycline	23.40 ± 1.20	31.50 ± 1.33	28.50 ± 1.47	41.55 ± 1.94	37.35 ± 0.63	24.85 ± 0.24	20.02 ± 0.33	18.65 ± 0.41	13.00 ± 0.41
	Erythromycin	23.25 ± 1.16	28.75 ± 0.49	24.20 ± 0.42	37.30 ± 0.67	33.20 ± 0.63	23.55 ± 0.60	21.65 ± 0.34	18.80 ± 1.87	5.90 ± 0.66
Enzymatic hydrolysis with *Viscozyme L*	n/a	17.95 ± 1.26	25.65 ± 0.82	17.55 ± 0.64	23.50 ± 0.62	18.75 ± 1.01	15.20 ± 0.48	12.95 ± 1.04	13.00 ± 0.82	11.35 ± 0.53
	Ceftazidime	31.25 ± 1.18	35.15 ± 0.67	30.70 ± 0.79	45.90 ± 1.20	34.20 ± 0.48	22.05 ± 1.46	19.20 ± 0.75	24.35 ± 1.25	15.80 ± 2.45
	Ciprofloxacin	26.50 ± 1.05	34.55 ± 0.83	30.75 ± 0.86	35.50 ± 1.86	28.20 ± 1.64	21.00 ± 0.62	20.50 ± 2.26	25.95 ± 1.32	14.45 ± 1.19
	Oxytetracycline	29.85 ± 1.27	37.80 ± 1.64	32.20 ± 1.09	38.25 ± 0.63	39.90 ± 2.72	27.35 ± 0.24	20.55 ± 1.09	19.20 ± 1.03	14.60 ± 1.43
	Erythromycin	28.50 ± 1.35	39.05 ± 1.04	33.45 ± 1.42	31.95 ± 0.90	33.05 ± 2.64	23.50 ± 0.02	19.75 ± 1.48	20.95 ± 0.16	6.75 ± 1.75
Enzymatic hydrolysis with amyloglucosidase	n/a	11.10 ± 0.84	13.80 ± 0.67	12.60 ± 0.66	17.95 ± 0.60	12.70 ± 0.48	10.75 ± 0.63	5.35 ± 1.13	6.05 ± 0.50	7.75 ± 0.86
	Ceftazidime	14.75 ± 0.42	18.80 ± 0.26	19.60 ± 0.74	25.50 ± 1.55	16.35 ± 0.24	16.35 ± 1.45	8.60 ± 0.32	6.85 ± 0.47	7.20 ± 0.42
	Ciprofloxacin	13.40 ± 0.57	19.50 ± 0.41	11.65 ± 0.24	24.10 ± 0.99	14.30 ± 0.54	13.90 ± 0.32	4.80 ± 0.48	6.25 ± 0.63	7.05 ± 0.44
	Oxytetracycline	17.15 ± 0.47	22.55 ± 0.60	28.05 ± 1.04	22.65 ± 0.75	20.80 ± 0.35	21.90 ± 1.02	13.90 ± 0.39	9.60 ± 0.52	5.25 ± 0.26
	Erythromycin	14.90 ± 0.70	20.90 ± 0.70	21.30 ± 0.54	19.10 ± 0.32	18.50 ± 0.58	17.05 ± 1.09	12.20 ± 0.54	18.30 ± 0.82	3.55 ± 0.16
**Antibacterial activity against *Listeria monocytogenes***										
Control	Ceftazidime	2.77 ± 0.20								
	Ciprofloxacin	2.96 ± 0.17								
	Oxytetracycline	3.21 ± 0.13								
	Erythromycin	3.75 ± 0.30								
Natural	n/a	6.32 ± 0.15	8.52 ± 0.15	8.52 ± 0.39	12.90 ± 0.39	10.67 ± 0.13	6.53 ± 0.16	5.34 ± 0.13	5.22 ± 0.14	5.10 ± 0.42
	Ceftazidime	19.30 ± 1.11	21.75 ± 0.63	18.85 ± 0.47	26.65 ± 0.24	23.40 ± 0.91	19.30 ± 0.82	14.65 ± 0.85	17.40 ± 0.70	13.60 ± 0.66

Treatment	Usage of antibiotic[1]	Bee pollen sample								
		Denmark	Sweden	Poland	Lithuania	Slovakia	Netherlands	Italy	Spain	Malta
	Ciprofloxacin	14.95 ± 0.60	17.05 ± 0.50	15.80 ± 0.82	20.02 ± 0.67	17.05 ± 0.83	9.80 ± 0.26	10.90 ± 1.20	12.20 ± 0.67	7.05 ± 0.69
	Oxytetracycline	24.70 ± 0.82	26.35 ± 1.62	24.65 ± 0.85	31.15 ± 1.20	28.35 ± 1.20	19.75 ± 1.32	18.55 ± 1.40	15.15 ± 0.71	11.90 ± 1.31
	Erythromycin	24.20 ± 2.18	28.35 ± 1.90	23.80 ± 1.11	37.50 ± 1.43	32.45 ± 1.04	23.05 ± 1.40	19.55 ± 1.77	17.55 ± 1.32	11.00 ± 1.55
Spontaneous fermentation	n/a	7.74 ± 0.10	11.46 ± 0.31	10.77 ± 0.23	14.27 ± 0.41	12.09 ± 0.48	8.01 ± 0.13	6.29 ± 0.27	7.20 ± 0.27	6.00 ± 0.47
	Ceftazidime	22.35 ± 1.00	25.25 ± 1.34	24.00 ± 0.85	29.30 ± 0.35	27.05 ± 0.80	22.40 ± 0.61	17.40 ± 0.88	18.90 ± 0.57	15.85 ± 0.75
	Ciprofloxacin	21.00 ± 0.88	22.25 ± 0.54	21.10 ± 0.61	25.15 ± 0.97	24.65 ± 0.85	20.75 ± 0.68	16.80 ± 1.46	19.20 ± 0.35	14.25 ± 0.86
	Oxytetracycline	27.95 ± 1.07	30.50 ± 1.20	28.40 ± 1.20	35.65 ± 1.83	30.05 ± 0.83	24.95 ± 0.55	19.90 ± 1.15	19.10 ± 0.46	16.30 ± 1.11
	Erythromycin	26.55 ± 1.55	35.50 ± 2.60	30.20 ± 1.48	39.65 ± 1.29	39.55 ± 1.06	29.50 ± 1.51	22.70 ± 1.86	23.80 ± 1.84	18.05 ± 1.23
Bacterial fermentation	n/a	9.32 ± 0.18	11.99 ± 0.17	11.04 ± 0.44	15.59 ± 0.11	13.17 ± 0.28	8.82 ± 0.09	7.20 ± 0.22	8.40 ± 0.38	6.54 ± 0.16
	Ceftazidime	23.40 ± 0.77	27.60 ± 0.32	25.40 ± 0.52	30.35 ± 1.11	27.05 ± 1.32	23.10 ± 0.70	17.55 ± 0.69	20.35 ± 0.82	17.65 ± 1.00
	Ciprofloxacin	22.00 ± 0.67	24.80 ± 1.25	24.05 ± 1.42	27.60 ± 0.70	25.80 ± 0.59	21.95 ± 0.64	17.65 ± 0.53	19.85 ± 0.67	15.40 ± 0.77
	Oxytetracycline	25.25 ± 2.03	31.50 ± 1.29	29.20 ± 1.46	35.95 ± 1.92	32.10 ± 0.88	26.60 ± 1.05	21.80 ± 0.89	22.45 ± 0.37	16.40 ± 0.81
	Erythromycin	27.95 ± 0.50	38.55 ± 1.92	34.70 ± 2.07	44.20 ± 2.18	42.85 ± 1.23	31.00 ± 2.20	23.80 ± 2.61	25.30 ± 1.67	20.65 ± 1.20
Enzymatic hydrolysis with protease	n/a	11.50 ± 0.91	10.30 ± 0.67	10.90 ± 1.66	14.80 ± 0.89	12.45 ± 1.59	11.85 ± 1.00	10.60 ± 1.17	10.40 ± 0.70	9.55 ± 0.96
	Ceftazidime	16.65 ± 0.34	14.20 ± 0.26	14.25 ± 0.26	15.80 ± 0.35	16.10 ± 0.77	13.20 ± 0.35	11.70 ± 0.75	13.15 ± 0.24	6.80 ± 0.35
	Ciprofloxacin	15.05 ± 0.76	9.60 ± 0.32	12.00 ± 0.47	15.75 ± 0.35	14.65 ± 0.47	9.00 ± 0.33	10.55 ± 0.28	11.80 ± 0.35	6.25 ± 0.42
	Oxytetracycline	18.85 ± 0.82	11.60 ± 0.21	16.20 ± 0.26	17.80 ± 0.26	17.25 ± 0.26	20.02 ± 1.03	17.00 ± 0.33	13.80 ± 0.26	8.05 ± 0.44
	Erythromycin	22.90 ± 0.52	16.30 ± 0.54	18.00 ± 0.78	22.95 ± 0.28	32.90 ± 1.17	22.55 ± 1.07	16.25 ± 0.86	16.25 ± 0.26	11.20 ± 0.42
Enzymatic hydrolysis with lipase	n/a	9.40 ± 1.22	9.35 ± 1.42	10.35 ± 1.11	13.85 ± 1.08	12.10 ± 1.70	10.35 ± 1.36	9.00 ± 1.08	9.65 ± 0.85	9.70 ± 0.67
	Ceftazidime	15.00 ± 0.71	9.90 ± 0.32	10.25 ± 0.26	15.35 ± 0.34	16.50 ± 0.24	8.85 ± 0.24	11.45 ± 0.16	10.25 ± 0.35	10.25 ± 0.59
	Ciprofloxacin	13.55 ± 0.16	10.02 ± 0.02	10.60 ± 0.21	15.90 ± 0.21	14.80 ± 0.42	9.00 ± 0.02	9.95 ± 0.16	12.10 ± 0.21	6.40 ± 0.32
	Oxytetracycline	16.55 ± 0.76	12.05 ± 0.16	17.00 ± 0.02	19.65 ± 0.41	19.65 ± 0.24	16.95 ± 0.37	10.30 ± 0.42	14.05 ± 0.28	8.40 ± 0.52
	Erythromycin	20.15 ± 0.75	15.95 ± 0.16	15.20 ± 0.26	26.85 ± 0.24	22.60 ± 0.57	18.70 ± 0.54	16.20 ± 0.67	10.70 ± 1.25	9.80 ± 0.92
Enzymatic hydrolysis with cellulase	n/a	14.15 ± 1.51	16.10 ± 1.13	26.65 ± 1.00	17.50 ± 1.22	15.95 ± 1.12	13.60 ± 0.81	17.70 ± 1.46	15.50 ± 1.22	13.55 ± 0.50
	Ceftazidime	23.15 ± 0.94	30.35 ± 0.41	27.00 ± 1.62	27.00 ± 1.11	25.45 ± 0.37	23.35 ± 0.71	25.30 ± 0.42	22.60 ± 0.74	27.70 ± 0.42
	Ciprofloxacin	23.45 ± 2.55	27.65 ± 1.38	25.80 ± 0.98	24.85 ± 0.82	25.85 ± 2.16	22.05 ± 0.72	24.90 ± 0.91	22.65 ± 0.71	25.35 ± 0.75
	Oxytetracycline	26.55 ± 1.12	36.00 ± 0.97	32.15 ± 1.06	31.50 ± 0.78	29.25 ± 0.75	25.35 ± 0.75	27.40 ± 0.46	26.85 ± 0.94	26.50 ± 0.85
	Erythromycin	30.95 ± 2.88	42.25 ± 0.68	41.80 ± 2.06	38.60 ± 1.96	34.80 ± 0.67	27.90 ± 1.56	30.30 ± 1.99	28.80 ± 1.32	30.75 ± 1.46
	n/a	13.15 ± 1.80	12.00 ± 0.97	12.30 ± 0.71	14.50 ± 1.37	12.90 ± 2.12	12.65 ± 0.71	13.15 ± 1.08	9.40 ± 0.81	12.05 ± 0.50

Treatment	Usage of antibiotic[1]	Bee pollen sample								
		Denmark	Sweden	Poland	Lithuania	Slovakia	Netherlands	Italy	Spain	Malta
Enzymatic hydrolysis with *Clara-diastase*	Ceftazidime	36.25 ± 48.95	22.15 ± 0.47	22.55 ± 4.12	32.70 ± 1.09	28.05 ± 0.86	22.40 ± 0.32	24.30 ± 0.79	25.45 ± 0.16	22.40 ± 0.97
	Ciprofloxacin	19.05 ± 2.14	20.10 ± 0.32	22.00 ± 1.62	29.80 ± 0.75	24.45 ± 0.16	20.80 ± 0.63	19.80 ± 0.79	17.80 ± 0.42	16.05 ± 0.37
	Oxytetracycline	26.50 ± 0.24	26.65 ± 0.67	25.60 ± 0.46	32.20 ± 0.26	32.35 ± 0.71	24.90 ± 0.52	29.80 ± 0.67	23.25 ± 0.92	20.45 ± 0.16
	Erythromycin	25.50 ± 1.00	34.75 ± 0.35	25.15 ± 0.75	44.85 ± 1.27	42.25 ± 0.98	29.45 ± 1.44	40.70 ± 0.67	23.70 ± 0.35	17.65 ± 0.47
Enzymatic hydrolysis with *Viscozyme L*	n/a	12.30 ± 1.38	15.65 ± 0.88	15.35 ± 2.03	15.95 ± 0.72	13.90 ± 0.91	12.55 ± 1.67	15.40 ± 0.97	13.25 ± 0.79	12.75 ± 0.59
	Ceftazidime	22.40 ± 0.46	29.25 ± 0.54	27.05 ± 0.50	37.85 ± 0.85	23.95 ± 0.86	22.40 ± 1.58	23.85 ± 0.34	23.20 ± 0.67	20.70 ± 1.01
	Ciprofloxacin	20.70 ± 1.11	25.10 ± 0.66	25.00 ± 1.25	23.95 ± 2.69	21.05 ± 0.76	20.95 ± 0.50	24.05 ± 0.50	21.20 ± 1.18	19.20 ± 1.11
	Oxytetracycline	24.80 ± 0.67	35.70 ± 1.55	29.95 ± 1.85	40.80 ± 1.23	28.45 ± 1.12	23.85 ± 1.06	24.15 ± 0.41	25.00 ± 1.11	21.25 ± 0.79
	Erythromycin	29.50 ± 2.59	43.85 ± 1.89	41.70 ± 2.04	44.50 ± 1.33	44.45 ± 1.69	26.40 ± 1.60	28.80 ± 1.14	27.65 ± 0.85	23.05 ± 1.89
Enzymatic hydrolysis with amyloglucosidase	n/a	10.50 ± 1.91	9.35 ± 1.45	9.25 ± 1.64	13.50 ± 0.58	12.05 ± 0.86	8.70 ± 0.67	8.90 ± 0.91	7.65 ± 0.78	8.00 ± 0.58
	Ceftazidime	14.85 ± 0.47	11.55 ± 0.16	14.30 ± 0.26	16.60 ± 1.71	11.00 ± 0.24	17.45 ± 1.28	13.75 ± 0.26	7.30 ± 0.26	7.40 ± 0.21
	Ciprofloxacin	13.50 ± 0.33	10.65 ± 0.34	10.90 ± 0.21	13.35 ± 1.73	11.30 ± 0.26	9.65 ± 0.24	6.55 ± 0.16	6.45 ± 0.37	6.90 ± 0.21
	Oxytetracycline	16.60 ± 0.88	15.90 ± 0.32	15.75 ± 0.26	19.90 ± 0.46	18.40 ± 0.46	18.20 ± 1.16	17.25 ± 0.79	12.35 ± 0.71	9.05 ± 0.16
	Erythromycin	20.20 ± 0.42	19.50 ± 0.41	23.55 ± 0.60	28.40 ± 0.77	24.25 ± 0.35	21.70 ± 0.54	18.75 ± 1.44	26.50 ± 1.43	10.25 ± 0.82
**Antibacterial activity against *Salmonella enterica* serovar Enteritidis**										
Control	Ceftazidime	2.93 ± 0.14								
	Ciprofloxacin	2.30 ± 0.07								
	Oxytetracycline	2.31 ± 0.06								
	Erythromycin	2.86 ± 0.12								
Natural	n/a	7.82 ± 0.57	10.40 ± 0.61	8.36 ± 0.17	15.31 ± 0.54	10.94 ± 0.19	7.04 ± 0.05	3.71 ± 0.66	4.46 ± 0.07	2.13 ± 0.46
	Ceftazidime	22.50 ± 1.78	28.95 ± 0.98	23.20 ± 1.40	36.00 ± 1.90	31.70 ± 1.89	12.15 ± 2.64	9.70 ± 2.07	9.95 ± 0.72	11.30 ± 0.86
	Ciprofloxacin	9.55 ± 1.01	21.65 ± 1.33	14.10 ± 1.41	24.25 ± 0.95	19.85 ± 1.47	8.75 ± 1.09	18.15 ± 1.00	7.95 ± 0.69	11.15 ± 0.85
	Oxytetracycline	14.90 ± 1.41	21.85 ± 1.13	24.95 ± 1.14	33.10 ± 1.51	30.40 ± 2.08	11.65 ± 0.53	19.85 ± 0.85	8.85 ± 1.94	11.10 ± 0.46
	Erythromycin	18.85 ± 2.07	24.65 ± 2.58	27.40 ± 2.39	33.45 ± 2.07	25.15 ± 1.51	13.20 ± 1.69	20.45 ± 1.69	9.30 ± 0.95	8.55 ± 1.85
Spontaneous fermentation	n/a	10.65 ± 0.41	12.48 ± 0.22	10.67 ± 0.64	18.95 ± 0.10	13.25 ± 0.66	9.89 ± 0.41	7.32 ± 0.46	9.89 ± 0.48	5.49 ± 0.93
	Ceftazidime	27.85 ± 2.12	34.15 ± 1.06	32.85 ± 1.42	36.70 ± 2.54	38.30 ± 2.21	31.70 ± 1.11	25.65 ± 1.53	28.40 ± 1.85	22.55 ± 1.21
	Ciprofloxacin	19.85 ± 1.11	26.15 ± 0.78	22.90 ± 1.07	28.20 ± 1.96	22.85 ± 1.00	12.95 ± 0.55	29.80 ± 0.92	16.35 ± 0.97	16.20 ± 0.54
	Oxytetracycline	27.05 ± 1.99	31.15 ± 0.88	32.40 ± 2.25	37.40 ± 0.88	34.95 ± 1.30	25.15 ± 1.23	24.45 ± 1.21	18.70 ± 1.03	14.90 ± 0.91
	Erythromycin	25.40 ± 2.46	27.45 ± 2.36	29.25 ± 1.84	36.50 ± 2.17	33.60 ± 0.88	23.95 ± 1.14	20.40 ± 1.29	22.00 ± 0.71	16.25 ± 2.95
	n/a	11.73 ± 0.14	14.30 ± 0.14	11.22 ± 0.06	21.54 ± 0.08	19.46 ± 0.14	11.21 ± 0.34	8.58 ± 0.09	10.44 ± 0.49	7.07 ± 1.25

Treatment	Usage of antibiotic[1]	Bee pollen sample								
		Denmark	Sweden	Poland	Lithuania	Slovakia	Netherlands	Italy	Spain	Malta
Bacterial fermentation	Ceftazidime	32.30 ± 2.02	36.60 ± 1.63	35.70 ± 1.11	45.25 ± 1.99	44.30 ± 1.48	38.05 ± 0.72	29.60 ± 1.20	31.05 ± 1.01	29.10 ± 0.57
	Ciprofloxacin	20.45 ± 1.12	28.00 ± 1.00	25.60 ± 1.74	35.10 ± 1.71	34.45 ± 1.57	29.40 ± 1.02	23.50 ± 1.33	26.50 ± 0.94	20.70 ± 0.98
	Oxytetracycline	28.60 ± 1.68	37.45 ± 1.26	33.45 ± 1.86	43.75 ± 1.78	39.20 ± 1.44	32.15 ± 0.78	26.05 ± 0.90	23.65 ± 1.25	20.10 ± 1.10
	Erythromycin	29.45 ± 1.54	36.45 ± 2.10	33.05 ± 1.28	41.30 ± 2.06	40.85 ± 2.31	31.40 ± 1.61	23.55 ± 1.32	25.45 ± 1.48	18.90 ± 1.49
Enzymatic hydrolysis with protease	n/a	13.45 ± 2.59	12.05 ± 1.92	11.45 ± 2.61	16.60 ± 1.15	12.70 ± 1.40	8.40 ± 0.61	7.30 ± 0.54	7.30 ± 1.23	7.25 ± 2.43
	Ceftazidime	20.45 ± 1.76	16.90 ± 0.46	13.35 ± 0.24	18.00 ± 0.53	15.00 ± 2.08	7.95 ± 0.28	10.10 ± 0.61	7.80 ± 0.26	5.65 ± 0.24
	Ciprofloxacin	9.40 ± 0.88	9.65 ± 0.34	11.60 ± 0.70	15.00 ± 0.02	11.50 ± 0.62	7.20 ± 0.75	16.15 ± 0.67	5.00 ± 0.02	2.40 ± 0.32
	Oxytetracycline	11.45 ± 1.09	15.40 ± 0.52	9.65 ± 0.47	16.40 ± 0.39	14.90 ± 1.56	12.20 ± 0.63	15.30 ± 0.26	8.55 ± 0.16	5.55 ± 1.17
	Erythromycin	16.35 ± 1.84	12.40 ± 0.84	11.05 ± 0.83	17.30 ± 0.75	16.20 ± 3.15	10.05 ± 0.60	12.65 ± 0.24	7.90 ± 0.21	3.10 ± 0.57
Enzymatic hydrolysis with lipase	n/a	8.50 ± 2.05	11.50 ± 1.76	10.05 ± 1.86	15.95 ± 0.80	11.00 ± 2.40	8.00 ± 1.22	6.15 ± 0.97	5.30 ± 1.21	5.65 ± 1.38
	Ceftazidime	15.90 ± 0.21	12.15 ± 0.24	10.50 ± 0.71	17.85 ± 0.34	21.95 ± 0.16	10.80 ± 0.48	6.00 ± 0.02	6.50 ± 0.02	5.60 ± 1.29
	Ciprofloxacin	5.45 ± 0.16	8.70 ± 0.35	9.45 ± 0.72	14.35 ± 0.58	15.45 ± 0.37	6.00 ± 0.33	8.90 ± 0.21	7.00 ± 0.41	9.90 ± 0.66
	Oxytetracycline	8.35 ± 0.24	11.55 ± 0.28	25.05 ± 0.86	16.85 ± 0.24	19.15 ± 0.24	7.75 ± 0.63	7.35 ± 0.58	5.50 ± 0.02	7.30 ± 0.26
	Erythromycin	18.85 ± 2.03	12.55 ± 0.37	25.65 ± 1.03	19.20 ± 0.26	17.45 ± 0.16	13.00 ± 0.41	10.05 ± 0.28	7.80 ± 0.42	5.40 ± 0.21
Enzymatic hydrolysis with cellulase	n/a	17.45 ± 0.93	18.90 ± 1.02	21.90 ± 1.82	22.90 ± 1.35	23.35 ± 1.13	17.20 ± 1.42	12.20 ± 0.92	13.95 ± 1.09	12.90 ± 1.73
	Ceftazidime	32.85 ± 0.94	42.00 ± 1.65	36.85 ± 0.71	36.70 ± 1.38	35.75 ± 0.79	32.05 ± 3.10	23.15 ± 1.96	26.35 ± 4.78	27.15 ± 4.92
	Ciprofloxacin	23.20 ± 1.77	29.15 ± 0.58	27.00 ± 0.94	28.00 ± 0.62	28.25 ± 2.72	22.20 ± 2.90	25.20 ± 0.71	19.50 ± 1.00	19.45 ± 0.28
	Oxytetracycline	24.40 ± 0.39	33.05 ± 1.32	31.40 ± 1.07	41.65 ± 2.24	37.05 ± 0.98	23.85 ± 0.82	23.60 ± 1.05	26.10 ± 0.91	20.10 ± 0.21
	Erythromycin	26.95 ± 3.19	40.30 ± 1.44	42.90 ± 1.73	41.20 ± 0.67	31.95 ± 2.63	25.60 ± 2.34	25.45 ± 1.54	23.45 ± 1.04	18.95 ± 1.66
Enzymatic hydrolysis with *Clara-diastase*	n/a	12.15 ± 2.14	17.00 ± 1.75	18.60 ± 1.51	16.70 ± 1.36	16.10 ± 0.88	10.60 ± 2.39	11.05 ± 2.11	11.25 ± 0.54	10.65 ± 0.85
	Ceftazidime	28.90 ± 0.84	31.15 ± 0.24	36.95 ± 2.34	40.10 ± 1.70	32.30 ± 0.67	25.70 ± 1.14	22.55 ± 1.66	23.15 ± 0.34	23.90 ± 4.45
	Ciprofloxacin	19.80 ± 4.72	23.20 ± 1.55	25.20 ± 1.57	28.00 ± 0.02	23.70 ± 0.48	15.95 ± 4.30	19.20 ± 1.53	18.50 ± 0.41	20.55 ± 0.50
	Oxytetracycline	22.00 ± 1.47	33.00 ± 0.02	35.65 ± 1.27	37.35 ± 0.34	30.60 ± 0.32	17.05 ± 1.55	22.95 ± 0.76	18.05 ± 0.44	11.90 ± 1.35
	Erythromycin	23.85 ± 2.11	27.75 ± 2.15	29.75 ± 0.86	37.00 ± 0.67	41.30 ± 1.32	18.80 ± 3.39	31.80 ± 1.99	20.45 ± 0.37	11.50 ± 1.80
Enzymatic hydrolysis with *Viscozyme L*	n/a	14.45 ± 1.95	18.15 ± 2.26	20.45 ± 2.35	21.30 ± 1.74	17.40 ± 1.87	15.15 ± 2.37	11.30 ± 1.86	9.75 ± 1.59	11.35 ± 2.73
	Ceftazidime	31.80 ± 1.27	36.60 ± 2.46	38.35 ± 1.97	43.45 ± 1.82	32.85 ± 3.24	27.85 ± 2.95	28.35 ± 1.96	25.65 ± 0.82	25.55 ± 5.10
	Ciprofloxacin	20.70 ± 5.32	28.25 ± 1.30	22.85 ± 1.75	26.25 ± 1.32	22.85 ± 0.85	19.85 ± 1.31	24.55 ± 1.99	14.50 ± 0.82	18.30 ± 5.22
	Oxytetracycline	25.20 ± 1.93	37.40 ± 1.73	32.95 ± 1.40	37.20 ± 0.63	32.45 ± 1.12	23.15 ± 2.12	28.15 ± 2.54	24.35 ± 1.65	17.45 ± 4.11
	Erythromycin	25.60 ± 0.66	36.55 ± 1.01	33.50 ± 2.43	35.65 ± 2.07	26.70 ± 1.64	25.45 ± 2.69	22.00 ± 0.47	20.45 ± 0.76	19.50 ± 4.20

Treatment	Usage of antibiotic[1]	Bee pollen sample								
		Denmark	Sweden	Poland	Lithuania	Slovakia	Netherlands	Italy	Spain	Malta
Enzymatic hydrolysis with amyloglucosidase	n/a	7.40 ± 1.13	11.15 ± 2.20	8.80 ± 1.48	16.00 ± 1.47	11.90 ± 2.25	7.90 ± 1.45	4.70 ± 1.44	5.10 ± 0.99	4.85 ± 1.33
	Ceftazidime	18.25 ± 1.03	17.30 ± 0.48	9.80 ± 0.63	25.65 ± 2.20	12.60 ± 0.52	11.25 ± 0.26	8.35 ± 0.34	7.10 ± 0.70	11.05 ± 0.37
	Ciprofloxacin	9.15 ± 0.63	12.10 ± 0.21	9.70 ± 0.59	16.95 ± 1.69	10.60 ± 0.39	6.35 ± 0.47	3.60 ± 0.32	4.85 ± 0.58	9.00 ± 0.02
	Oxytetracycline	13.10 ± 0.57	11.55 ± 0.76	10.20 ± 0.35	17.05 ± 0.83	17.30 ± 0.35	11.45 ± 0.50	20.20 ± 0.63	5.80 ± 0.67	5.55 ± 0.44
	Erythromycin	18.70 ± 1.38	8.45 ± 0.28	21.80 ± 0.35	19.50 ± 0.02	18.95 ± 0.37	12.55 ± 0.44	13.25 ± 1.27	6.40 ± 0.21	7.45 ± 0.16
**Antibacterial activity against *Salmonella enterica* serovar Typhimurium**										
Control	Ceftazidime	3.34 ± 0.44								
	Ciprofloxacin	3.28 ± 0.34								
	Oxytetracycline	3.40 ± 0.50								
	Erythromycin	3.05 ± 0.57								
Natural	n/a	2.60 ± 0.10	6.14 ± 0.13	5.94 ± 0.18	9.14 ± 0.27	6.50 ± 0.16	2.45 ± 0.16	1.23 ± 0.15	1.08 ± 0.15	0.91 ± 0.25
	Ceftazidime	12.50 ± 0.02	16.15 ± 0.94	15.45 ± 0.86	20.80 ± 1.06	13.65 ± 0.63	9.30 ± 1.27	6.80 ± 1.01	6.00 ± 0.58	5.15 ± 0.34
	Ciprofloxacin	11.00 ± 0.02	15.70 ± 0.98	15.20 ± 0.42	17.40 ± 1.07	11.20 ± 0.59	11.85 ± 0.82	6.90 ± 0.84	6.90 ± 0.77	6.30 ± 1.11
	Oxytetracycline	15.40 ± 0.91	22.60 ± 1.60	16.95 ± 0.80	22.35 ± 0.94	20.75 ± 0.54	14.50 ± 1.49	9.65 ± 1.13	7.90 ± 0.61	8.25 ± 1.14
	Erythromycin	10.95 ± 1.17	23.40 ± 2.00	19.75 ± 0.72	25.90 ± 1.74	24.35 ± 0.53	14.95 ± 1.79	11.35 ± 0.91	10.25 ± 1.21	8.85 ± 1.67
Spontaneous fermentation	n/a	3.71 ± 0.53	7.34 ± 0.15	6.53 ± 0.33	10.56 ± 1.02	7.47 ± 0.18	4.02 ± 0.32	2.15 ± 0.45	3.15 ± 0.47	1.52 ± 0.37
	Ceftazidime	16.65 ± 0.63	19.30 ± 1.23	19.80 ± 1.25	27.05 ± 1.52	21.60 ± 1.39	14.35 ± 1.38	13.65 ± 0.47	13.20 ± 1.97	9.80 ± 0.95
	Ciprofloxacin	15.35 ± 1.08	17.55 ± 1.21	18.65 ± 0.58	22.65 ± 0.97	19.30 ± 1.38	17.55 ± 1.12	11.85 ± 1.33	13.25 ± 1.23	8.95 ± 0.93
	Oxytetracycline	22.80 ± 1.84	24.95 ± 1.80	23.75 ± 0.63	28.20 ± 2.20	27.70 ± 1.03	19.00 ± 1.15	15.35 ± 1.70	17.05 ± 2.03	13.55 ± 1.23
	Erythromycin	17.35 ± 1.62	29.35 ± 1.58	26.95 ± 1.48	37.85 ± 1.65	30.80 ± 0.89	23.80 ± 1.06	16.05 ± 2.57	19.75 ± 1.42	9.30 ± 1.78
Bacterial fermentation	n/a	5.40 ± 0.40	8.18 ± 0.23	7.19 ± 0.13	11.94 ± 0.28	8.21 ± 0.37	4.83 ± 0.15	2.75 ± 0.22	4.41 ± 0.08	1.62 ± 0.09
	Ceftazidime	22.15 ± 0.63	27.05 ± 0.80	25.30 ± 1.67	30.50 ± 0.53	25.05 ± 1.07	15.70 ± 0.63	14.40 ± 1.76	15.45 ± 0.60	12.50 ± 0.58
	Ciprofloxacin	22.30 ± 0.54	26.30 ± 0.67	25.65 ± 0.53	29.35 ± 0.75	26.55 ± 0.80	20.75 ± 0.72	13.35 ± 0.71	15.15 ± 0.91	10.95 ± 1.07
	Oxytetracycline	26.05 ± 1.09	31.35 ± 0.91	30.15 ± 0.91	36.70 ± 0.79	32.35 ± 1.08	24.90 ± 1.51	17.15 ± 1.11	21.25 ± 0.98	22.65 ± 1.55
	Erythromycin	25.15 ± 1.45	36.70 ± 1.16	33.20 ± 1.78	41.85 ± 1.13	38.85 ± 2.69	29.30 ± 2.65	18.60 ± 1.33	22.65 ± 1.03	10.90 ± 1.31
Enzymatic hydrolysis with protease	n/a	5.75 ± 2.02	10.15 ± 2.11	8.05 ± 1.26	11.45 ± 3.16	9.45 ± 2.17	8.70 ± 1.49	7.35 ± 0.97	5.55 ± 1.69	3.50 ± 0.97
	Ceftazidime	9.85 ± 0.47	14.10 ± 0.21	7.65 ± 0.58	10.70 ± 0.42	8.05 ± 0.37	9.10 ± 0.32	6.40 ± 0.99	3.95 ± 0.16	4.60 ± 0.84
	Ciprofloxacin	6.40 ± 0.39	7.35 ± 0.34	7.45 ± 0.16	15.95 ± 0.16	8.75 ± 0.68	11.85 ± 0.71	6.10 ± 0.70	4.10 ± 0.21	2.65 ± 0.63
	Oxytetracycline	8.25 ± 0.82	10.95 ± 0.16	10.45 ± 0.28	19.50 ± 0.02	11.75 ± 0.26	9.45 ± 0.37	8.50 ± 0.02	6.80 ± 0.26	3.15 ± 0.34
	Erythromycin	10.80 ± 0.26	12.20 ± 0.48	10.95 ± 0.50	16.15 ± 0.47	18.60 ± 1.17	11.00 ± 1.72	11.25 ± 0.63	6.80 ± 0.26	3.95 ± 0.44

Treatment	Usage of antibiotic[1]	Bee pollen sample								
		Denmark	Sweden	Poland	Lithuania	Slovakia	Netherlands	Italy	Spain	Malta
Enzymatic hydrolysis with lipase	n/a	4.75 ± 1.11	7.35 ± 2.30	7.65 ± 2.03	9.75 ± 2.37	9.15 ± 1.36	6.05 ± 1.44	5.80 ± 1.03	4.50 ± 1.58	3.35 ± 0.75
	Ceftazidime	9.30 ± 0.48	6.60 ± 0.32	7.50 ± 1.37	18.45 ± 0.44	13.45 ± 0.37	4.45 ± 0.16	5.45 ± 0.16	3.05 ± 0.16	2.50 ± 0.02
	Ciprofloxacin	12.30 ± 0.54	8.60 ± 0.21	7.75 ± 0.35	15.95 ± 0.37	12.40 ± 0.21	11.45 ± 0.76	5.10 ± 0.21	6.50 ± 0.33	2.55 ± 0.37
	Oxytetracycline	14.05 ± 0.50	9.75 ± 0.26	12.30 ± 1.11	14.30 ± 0.59	15.55 ± 0.16	12.05 ± 0.28	5.15 ± 0.34	4.65 ± 0.24	3.90 ± 0.61
	Erythromycin	21.15 ± 0.71	15.05 ± 0.44	24.95 ± 0.86	16.95 ± 0.50	16.25 ± 0.72	9.65 ± 0.82	7.15 ± 0.63	4.45 ± 1.01	3.50 ± 0.41
Enzymatic hydrolysis with cellulase	n/a	9.00 ± 2.17	11.95 ± 1.46	14.00 ± 1.27	18.50 ± 2.15	13.90 ± 1.68	14.05 ± 2.47	9.30 ± 1.27	6.15 ± 1.11	5.75 ± 0.95
	Ceftazidime	15.60 ± 1.24	22.60 ± 0.32	22.95 ± 1.34	26.90 ± 0.61	23.20 ± 1.11	22.15 ± 0.82	16.55 ± 2.65	14.40 ± 1.22	12.45 ± 0.93
	Ciprofloxacin	20.65 ± 0.53	23.90 ± 0.99	21.20 ± 0.89	23.20 ± 0.79	21.45 ± 2.09	22.35 ± 1.27	15.65 ± 1.83	13.30 ± 1.70	11.90 ± 1.51
	Oxytetracycline	21.10 ± 0.77	29.55 ± 0.76	25.75 ± 1.09	24.80 ± 0.48	24.85 ± 0.71	21.40 ± 1.68	19.30 ± 0.63	17.15 ± 0.94	16.00 ± 0.75
	Erythromycin	24.95 ± 1.98	38.15 ± 1.08	38.85 ± 0.94	28.40 ± 0.99	27.30 ± 0.89	25.15 ± 1.45	22.65 ± 0.75	18.60 ± 1.88	18.00 ± 0.58
Enzymatic hydrolysis with *Clara-diastase*	n/a	7.90 ± 1.73	10.90 ± 1.26	12.10 ± 2.67	15.45 ± 0.93	10.75 ± 1.36	10.80 ± 1.83	7.35 ± 2.33	5.05 ± 0.72	4.25 ± 1.21
	Ceftazidime	19.30 ± 1.30	21.45 ± 0.16	22.20 ± 1.78	28.40 ± 0.88	18.40 ± 0.66	13.55 ± 1.36	15.55 ± 5.00	9.00 ± 2.16	12.95 ± 2.14
	Ciprofloxacin	17.55 ± 0.76	18.60 ± 0.21	21.30 ± 2.84	39.85 ± 0.24	18.30 ± 0.35	15.45 ± 0.55	14.70 ± 4.30	11.60 ± 3.23	11.90 ± 3.78
	Oxytetracycline	18.15 ± 1.81	25.70 ± 0.26	26.45 ± 1.26	43.10 ± 0.21	21.90 ± 0.46	18.55 ± 1.17	15.30 ± 1.62	13.00 ± 4.86	10.75 ± 1.92
	Erythromycin	23.40 ± 0.74	29.60 ± 1.07	27.35 ± 0.71	41.95 ± 1.42	26.60 ± 1.20	29.95 ± 0.50	22.35 ± 2.10	16.65 ± 0.34	10.80 ± 2.35
Enzymatic hydrolysis with *Viscozyme L*	n/a	8.40 ± 1.87	11.50 ± 1.37	13.65 ± 1.62	17.50 ± 1.83	11.45 ± 0.93	10.80 ± 1.27	8.00 ± 2.20	5.20 ± 0.89	4.90 ± 0.88
	Ceftazidime	17.35 ± 3.04	27.05 ± 0.93	22.60 ± 2.08	45.40 ± 1.33	19.80 ± 0.89	17.45 ± 2.40	14.15 ± 4.81	13.60 ± 0.94	9.75 ± 1.16
	Ciprofloxacin	17.60 ± 0.61	23.25 ± 1.18	22.45 ± 3.53	43.60 ± 0.66	18.65 ± 0.47	18.10 ± 3.33	13.35 ± 0.63	11.85 ± 0.47	8.95 ± 0.98
	Oxytetracycline	18.95 ± 0.28	28.25 ± 0.26	27.65 ± 0.53	24.95 ± 1.07	23.70 ± 1.34	17.65 ± 2.71	16.95 ± 1.30	15.35 ± 2.00	13.70 ± 1.03
	Erythromycin	23.80 ± 0.71	37.10 ± 0.32	30.75 ± 2.06	29.30 ± 0.59	24.40 ± 1.47	17.40 ± 2.55	19.65 ± 2.10	16.10 ± 3.33	9.35 ± 1.53
Enzymatic hydrolysis with amyloglucosidase	n/a	4.15 ± 1.13	6.50 ± 0.88	6.85 ± 1.42	9.55 ± 1.76	8.25 ± 1.70	5.50 ± 0.78	3.85 ± 0.78	2.00 ± 0.75	0.95 ± 0.44
	Ceftazidime	12.40 ± 0.57	6.55 ± 0.16	10.60 ± 0.57	10.60 ± 0.39	7.45 ± 0.16	6.30 ± 0.82	6.70 ± 0.48	4.50 ± 0.02	1.85 ± 0.47
	Ciprofloxacin	11.05 ± 0.64	10.45 ± 0.16	9.70 ± 0.26	12.95 ± 0.60	8.15 ± 0.71	8.20 ± 0.48	2.65 ± 0.24	4.25 ± 0.35	5.05 ± 0.16
	Oxytetracycline	12.00 ± 0.02	11.80 ± 0.42	28.35 ± 1.25	14.90 ± 1.60	15.10 ± 0.74	8.95 ± 0.69	6.60 ± 0.70	4.35 ± 0.34	4.50 ± 0.02
	Erythromycin	10.55 ± 0.37	18.70 ± 0.48	11.35 ± 0.71	17.30 ± 0.42	17.75 ± 0.26	10.90 ± 0.57	10.65 ± 0.82	8.80 ± 0.63	6.35 ± 0.24

[1] — n/a – bee pollen extract without added antibiotic.
